# Stem Cell-Derived Extracellular Vesicle-Mediated Therapeutic Signaling in Spinal Cord Injury

**DOI:** 10.3390/ijms26020723

**Published:** 2025-01-16

**Authors:** Raju Poongodi, Yung-Wei Hsu, Tao-Hsiang Yang, Ya-Hsien Huang, Kuender D. Yang, Hsin-Chieh Lin, Jen-Kun Cheng

**Affiliations:** 1 Department of Medical Research, MacKay Memorial Hospital, Taipei 10449, Taiwan; poongodiraju@gmail.com (R.P.); konnankonnan@yahoo.com.tw (T.-H.Y.); 2 Department of Anesthesiology, MacKay Memorial Hospital, Taipei 10449, Taiwan; yungweih@gmail.com (Y.-W.H.); trista468@gmail.com (Y.-H.H.); 3 Department of Medicine, MacKay Medical College, New Taipei City 25245, Taiwan; 4 Institute of Long-Term Care, MacKay Medical College, New Taipei City 25245, Taiwan; yangkd.yeh@hotmail.com; 5 MacKay Children’s Hospital, Taipei 10449, Taiwan; 6 Institute of Clinical Medicine, National Yang Ming Chiao Tung University, Taipei 11221, Taiwan; 7 Department of Materials Science and Engineering, National Yang Ming Chiao Tung University, Hsinchu 300093, Taiwan; hclin45@nycu.edu.tw; 8 Center for Intelligent Drug Systems and Smart Bio-Devices (IDS2B), National Yang Ming Chiao Tung University, Hsinchu 30068, Taiwan

**Keywords:** spinal cord injury, mesenchymal stem cell, extracellular vesicle, miRNA, signaling pathway, therapeutic effects, bio-scaffold

## Abstract

Mesenchymal stem cell-derived extracellular vesicles (MSC-EVs) have emerged as a promising therapeutic strategy for spinal cord injury (SCI). These nanosized vesicles possess unique properties such as low immunogenicity and the ability to cross biological barriers, making them ideal carriers for delivering bioactive molecules to injured tissues. MSC-EVs have been demonstrated to exert multiple beneficial effects in SCI, including reducing inflammation, promoting neuroprotection, and enhancing axonal regeneration. Recent studies have delved into the molecular mechanisms underlying MSC-EV-mediated therapeutic effects. Exosomal microRNAs (miRNAs) have been identified as key regulators of various cellular processes involved in SCI pathogenesis and repair. These miRNAs can influence inflammation, oxidative stress, and apoptosis by modulating gene expression. This review summarized the current state of MSC-EV-based therapies for SCI, highlighting the underlying mechanisms and potential clinical applications. We discussed the challenges and limitations of translating these therapies into clinical practice, such as inconsistent EV production, complex cargo composition, and the need for targeted delivery strategies. Future research should focus on optimizing EV production and characterization, identifying key therapeutic miRNAs, and developing innovative delivery systems to maximize the therapeutic potential of MSC-EVs in SCI.

## 1. Introduction

Spinal cord injury (SCI) is a devastating condition with limited therapeutic options, often leading to long-term disability and impaired quality of life [1]. Repairing SCI involves addressing complex pathophysiological mechanisms and complications related to nerve regeneration within the nervous system [2]. The etiology of SCI encompasses primary and secondary injuries, initially manifesting as mechanical damage to the spinal cord, followed by the influx of cells and their biological reactions to the primary injury [3]. This process engages various systems, including the nervous, immune, and vascular systems, resulting in inflammatory responses, scar formation, neural cell death, demyelination, ischemia, oxidative stress, hemorrhage, etc. Repairing the damaged spinal cord is significantly challenging, with several approaches available for promoting neuroprotection, angiogenesis, immunomodulation, and axonal regeneration [4,5].

**Figure 1:**
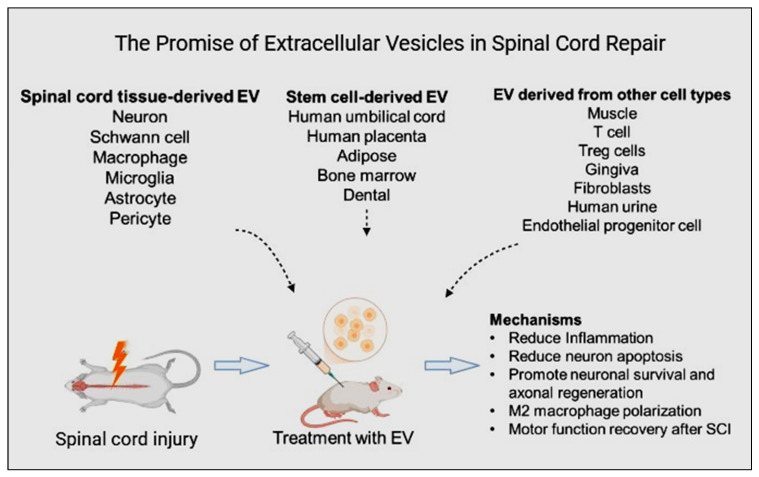
Illustration of extracellular vesicle-mediated therapeutic mechanisms for spinal cord injury repair. Various types of extracellular vesicles, including mesenchymal stem cell-derived exosomes, neuronal microvesicles, and macrophage-derived exosomes, hold promise for spinal cord repair. These vesicles can exert therapeutic effects by reducing inflammation, promoting axonal regeneration, and enhancing neurogenesis (created with BioRender.com).

The therapeutic benefits of MSCs in treating SCI are attributed to their paracrine mechanism supported by both in vivo and in vitro studies in rat SCI [6]. As shown in Figure 1, MSC-secreted nanosized EXs can potentially decrease inflammation and cell death, promote angiogenesis, and facilitate functional behavioral recovery in rat SCI [7,8,9]. Also, MSCs have a favorable safety profile in clinical trials for various diseases and are significant producers of immunologically inert exosomes, as noted in [10]. In addition, EVs hold promise for repairing injured spinal cord tissues through several mechanisms: angiogenesis [11] and axon formation [12], the regulation of inflammation and the immune response [13], the inhibition of apoptosis [14], and the maintenance of the BSCB’s integrity [15]. MSC-EXs may thus be a promising cell-free therapeutic approach for SCI treatment, mainly because of their paracrine effects as evidenced by studies in rat SCI [6] and in vitro cell culture experiments [7]. The limitations of existing therapeutic options for SCI highlight the necessity for innovative treatment strategies. This review article examined the preparation, functions, mechanisms, and challenges associated with different EVs in SCI treatment.

## 2. Extracellular Vesicles: Potential Mediators in Spinal Cord Injury

Extracellular vesicles (EVs) are emerging as a promising therapeutic strategy in regenerative medicine, including SCI [16,17]. These nanosized, membrane-bound particles, ranging from 40 to 1000 nanometers in diameter, are released by various cell types and carry diverse bioactive molecules [18]. Exosomes, a specific subtype of EVs originating from the endocytic pathway, are particularly enriched in proteins, lipids, and nucleic acids. These molecules can influence cellular behavior, promote tissue repair, and modulate immune responses. Given their potential to target injured tissues and deliver therapeutic payloads, EVs offer a promising avenue for developing innovative treatments for SCI. While EVs have gained significant attention in scientific research, a comprehensive bibliometric analysis of their role in SCI is lacking. To address this gap, this study aimed to systematically analyze the therapeutic effects of mechanisms related to EVs and SCI.

Exosomes (EXs), typically 30–150 nm in diameter, are continuously released into the extracellular environment and contain diverse biomolecules, including lipids, proteins, and nucleic acid [19]. These are crucial for intercellular communication because they carry a diverse array of active components, including proteins (e.g., TSG101, AIP1/ALIX, 1-integrin, CD81, CD63, ICAM-1, and MFG-E8), lipids (e.g., cholesterol, phosphatidylserine, phosphatidylinositol, sphingomyelin, and phosphatidylcholine), and nucleic acids (e.g., mRNA, miRNA, noncoding RNAs, and DNA) [20,21]. EXs are signaling elements released naturally from a mesenchymal stem cell (MSC), facilitating cell-to-cell communication by transporting genetic materials and proteins to the recipient cells, thereby initiating beneficial processes [22]. Notably, neurons, glial, and immune cells can also produce EXs to regulate biological processes following nerve injury [23]. 

The protein cargo of EVs exhibits notable differences between healthy and injured individuals, highlighting its potential as a crucial biomarker in serum analysis [24]. In healthy subjects, EVs primarily carry proteins essential for cellular homeostasis, including housekeeping proteins, structural proteins, and signaling molecules [24]. In addition, the immune regulatory proteins are abundant, maintaining immune tolerance [25]. In contrast, injured subjects exhibit elevated levels of inflammatory proteins (cytokines, chemokines, and adhesion molecules) within their EVs, potentially exacerbating tissue damage [24,26]. The levels of proteins involved in tissue repair and regeneration may be altered or decreased, hindering the healing process [27]. Furthermore, EVs from injured subjects often contain elevated levels of damage-associated molecular patterns (DAMPs), which can trigger and amplify inflammation [28]. These distinct protein profiles in EVs have significant implications for disease diagnosis, prognosis, and the development of novel therapeutic strategies.

Proteomic analyses of SCI tissue revealed intricate molecular changes, including alterations in proteins involved in inflammation, cell death, oxidative stress, neurodegeneration, lipid metabolism, and lysosomal function [29]. These studies identified a significant upregulation of proteins involved in lipid transport and cholesterol metabolism, such as APOE, NPC2, and ABCA1, as well as lysosomal proteins like cathepsins, crucial for cellular processes and implicated in neuroinflammation [30]. Concurrently, research demonstrated the therapeutic potential of EVs in SCI. EVs derived from various sources, including stem cells, exhibit neuroprotective, anti-inflammatory, and regenerative properties, promoting axonal growth and functional recovery [31]. Furthermore, engineered EVs carrying therapeutic cargos, such as miRNAs, offer promising strategies for the targeted modulation of gene expression and enhanced SCI repair [32]. These findings underscored the value of proteomic analysis for understanding SCI pathophysiology and highlighted the promising therapeutic potential of EVs for spinal cord repair.

## 3. Extraction and Identification of Extracellular Vesicles

**Figure 2:**
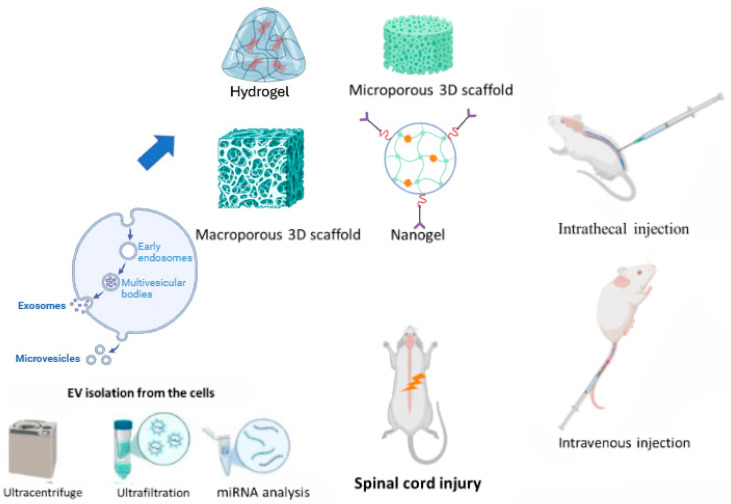
An overview of extracellular vesicle (EV) isolation, their miRNA analysis, and application of various bio-scaffolds and injections (created with BioRender.com).

The isolation of EV from biological sources like body fluids and cell cultures can vary depending on the exosome origin and size. Five primary isolation methods were documented: ultracentrifugation, size-based separation, in situ polymer precipitation, immunoaffinity capture, and microfluidic approaches [33]. Ultracentrifugation currently dominates as the most frequent method, as shown in Figure 2 [34]. While various extraction techniques exist, they often have limitations like low yield and insufficient purity. Furthermore, characterizing EV involves a multifaceted approach [35]. Techniques for identifying and analyzing exosomes encompass morphological assessments (transmission electron microscopy, scanning electron microscopy, cryo-electron microscopy, and atomic force microscopy), particle-size determination (nanoparticle tracking analysis and dynamic light scattering), surface marker analysis (trypsin digestion, mass spectrometry, and enzyme-linked immunosorbent assay (ELISA)), and protein expression profiling (Western blot analysis and flow cytometry) [34]. This necessitates further research into optimized extraction methods to enable large-scale exosome production for clinical applications.

## 4. Routes of Administration and Biomaterial Approaches for Extracellular Vesicle Delivery in Spinal Cord Injury

The administration routes for exosomes in rodent SCI models include intrathecal injection for direct delivery to the injury site, maximizing local effects and minimizing systemic side effects. Intravenous injections allow for systemic distribution, while epidural injection targets the spinal cord with fewer complications [36]. Compared to other routes like intrathecal injection, tail vein injection is generally less technically demanding and less likely to cause complications [37]. Tissue engineering has introduced biomaterial scaffolds (Figure 2) to deliver EXs. These scaffolds provide a supportive environment for EXs and stimulate neuronal axon growth [38]. At the SCI site, regenerative biomaterials can fill the cavities, deliver healing drugs, and provide adsorption sites for host cells [39]. Currently, the three main biomaterial scaffold types used for SCI are 3D-printed scaffolds, nanomaterial-based scaffolds, and biodegradable polymer scaffolds [39]. Moreover, the biodegradable polymer scaffolds, composed of agarose, chitosan, collagen, and fibronectin, were tested in animal models [40,41]. The collagen scaffold-encapsulated HucMSC-EXs can be designed using dual bio-specific peptides to help neural stem cell migration and paclitaxel delivery in SCI rats by promoting nerve regeneration, reducing scar deposition, and facilitating functional recovery [42]. In a rat model, EXs embedded with an alginate scaffold have anti-inflammatory, antinociceptive, and pro-neurotrophic properties in spinal nerve ligation injury-induced pain [43]. Overall, various administration routes for exosomes in SCI were explored, including intrathecal, intravenous, and epidural injections. Recent advancements in tissue engineering introduced novel approaches, such as incorporating exosomes within biomaterial scaffolds. These scaffolds, often composed of biodegradable polymers, provide a controlled release mechanism for exosomes, enhancing their therapeutic efficacy and promoting tissue regeneration.

## 5. Extracellular Vesicle miRNAs in Spinal Cord Injury Repair

MiRNAs regulate gene expression and play a unique role in nerve injury and regeneration [44]. Furthermore, exosomal miRNAs and neurotransmitter receptors can regulate synaptic transmission, neuronal excitability, and plasticity in the injured spinal cord, thereby preserving neuronal function and promoting functional recovery [45,46]. Moreover, stem cell EXs can stimulate endogenous neurogenesis [11] and oligodendrogenesis in the injured spinal cord by delivering trophic factors, extracellular matrix proteins, and developmental signaling molecules [47]. Additionally, miRNAs play a significant role in decreasing nerve apoptosis mediated via EXs. The distribution of miRNAs in SCI represents a promising therapeutic approach and can serve as potential biomarkers for nerve injuries [44]. Studies showed that specific miRNAs, such as miR-16-5p [48], miR-125b-5p [49,50], miR-21-5p [51], etc., are involved in regulating processes relevant to rat SCI. A previous review revealed miRNA transport via EXs in various cells and their significant protective effects in SCI [52]. Therefore, exosomal miRNAs exert a significant influence on neuronal function, regeneration, and survival following SCI. Understanding the complex interplay between miRNAs and exosomes provides crucial insights for developing novel therapeutic strategies to improve functional outcomes in patients with SCI.

## 6. Unlocking Therapeutic Potential: Mesenchymal Stem Cell-Derived Extracellular Vesicles in Spinal Cord Injury Recovery via Potential Signaling Pathways

### 6.1. Human Umbilical Cord Mesenchymal Stem Cell-Derived Extracellular Vesicles

Human umbilical cord mesenchymal stem cell-derived exosomes (HucMSC-EXs), the primary cell population neighboring the umbilical vessels, modulate antigen-presenting cells and T-cell apoptosis [53,54]. HucMSC-EXs mitigate apoptosis at the SCI site, downregulate inflammatory factors, and promote angiogenesis and axonal growth by activating the Wnt/β-catenin signaling pathway while simultaneously inhibiting microglia and astrocyte activation [54]. Moreover, HucMSC-EXs can induce the polarization of M1 macrophages toward the M2 phenotype [55]. HucMSC-EXs can reduce neuropathic pain, achieved by inhibiting the TLR2/MyD88/NF-κB signaling pathway in spinal microglia in a rat chronic constriction injury [56]. This effect involves the EX-mediated interference with Rsad2 expression, inhibiting microglial activation [56]. Additionally, the protective effects of HucMSC-EXs in rat SCI are mediated by the BCL2/Bax and Wnt/β-catenin signaling pathways [53]. Furthermore, EXs transfer miR-199a-3p/145-5p into neurons in SCI rats, affecting tropomyosin receptor kinase A (TrkA) ubiquitination and promoting the nerve growth factor (NGF)/TrkA signaling pathway [54].

HucMSC-EVs reduce pathological changes, enhance motor function, and promote nerve repair in SCI rats through the miR-29b-3p/Phosphatase and TENsin homolog deleted on chromosome 10 (PTEN)/Protein kinase B (PKB), also known as Akt/mammalian target of rapamycin (mTOR) pathway in rat SCI [57]. Additionally, miR-146a-5p within HucMSC-EXs can ameliorate the neuroinflammatory response mediated by microglia by suppressing the Interleukin-1 receptor-associated kinases 1 (IRAK1)/TNF receptor-associated factor (TRAF6) pathway in murine model [58]. Moreover, HucMSC-EXs effectively improves lipopolysaccharide/hydrogen peroxide-induced oxidative stress and neuroinflammation by inhibiting the microglial NRF2/NF-κB/NLRP3 signaling pathway in the LPS-treated mouse model [59]. Our recent article published that HucMSCs-EXs-encapsulated Gelfoam shows promise in improving motor dysfunction and neuropathic pain induced by SCI, possibly by promoting nerve regeneration, remyelination, anti-inflammatory processes, and anti-apoptotic mechanisms [1]. Therefore, HucMSCs-EXs are a powerful solution for repairing spinal cord injuries, making them a top choice for recovery.

### 6.2. Human Placental Mesenchymal Stem Cell-Derived Extracellular Vesicles

The therapeutic potential of human placental mesenchymal stem cell-derived exosomes (hPMSC-EXs) in rat SCI was demonstrated through their activation of endogenous neural pluripotent cells (NPCs) and facilitation of neurogenesis, leading to motor and autonomic function restoration [60]. This vital process may involve the activation of the mitogen-activated protein kinase (MEK)/extracellular signal-regulated kinase (ERK)/cAMP-response element binding protein (CREB) signaling pathway, which was implicated in alleviating rat nerve injury-induced neuropathic pain [60]. Furthermore, the synergistic effects of combining hPMSC-EXs treatment with hyperbaric oxygen enhance neuroprotective effects in SCI rats [61]. Moreover, the intrathecal injection of hPMSC-EXs restores function, neuronal regenerative capacity, and anti-apoptotic potential during the acute phase of SCI [61]. Additionally, miR-26a-5p within hPMSC-EVs can regulate the Wnt5a/Receptor-like Tyrosine Kinase (Ryk)/calmodulin-dependent kinase II (CaMKII)/nuclear factor of activated T cells (NFAT) pathways, offering moderate anti-neuroinflammatory and neuropathic pain relief effects through the Wnt signaling pathway in a spared nerve injury mouse model [62]. These findings provided strong evidence for the therapeutic potential of hPMSC-EXs in SCI. Their ability to promote neurogenesis, enhance neuroprotection, and alleviate neuropathic pain suggests that hPMSC-EXs may offer a novel and promising approach to treating SCI. Further research is warranted to translate these preclinical findings into effective clinical therapies.

### 6.3. Adipose Mesenchymal Stem Cell-Derived Extracellular Vesicles

Adipose-derived mesenchymal stem cells (ASCs) are a promising source of regenerative medicine due to their ease of isolation, high proliferation capacity, and immunomodulatory properties [63]. ASC-derived EXs are nanosized extracellular vesicles containing all neurotrophins, immunoregulatory, and angio-modulatory factors [63]. In vitro studies link ASC-EXs to a neuroprotective environment in neuronal differentiation and neuroregeneration. They stimulate PC12 cell migration/proliferation and inhibit apoptosis via PI3K/AKT pathway activation [64]. This effect could help treat nerve injuries, as ADSC-EXs were shown to promote significant repair of rat cavernous nerve injuries [65]. Hypoxia-conditioned ADSC-EXs significantly reduce neuronal apoptosis after the reperfusion (OGD/R) model in vitro [66]. Furthermore, it can reduce the formation of cavities in injured areas, leading to improved functional recovery of hind limbs in post-injury rats. The exosomal miR-499a-5p controls the JNK3/c-jun-apoptotic signaling pathway by affecting JNK3 and decreasing nerve apoptosis after rat SCI [66]. Furthermore, they promote functional recovery by reducing cavity formation in injured areas of rat SCI models. This neuroprotective effect is mediated by ADSC-EXs miR-499a-5p, which targets the JNK3/c-jun apoptotic signaling pathway, decreasing JNK3 expression and ultimately reducing nerve cell death after rat SCI [66].

**Figure 3:**
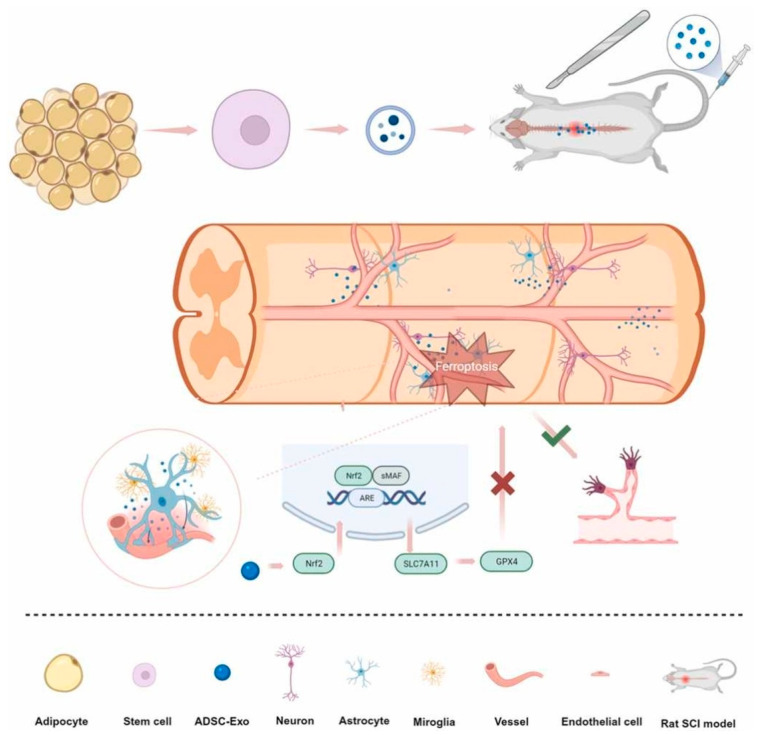
ADSC-EXs may inhibit ferroptosis and promote vascular and neural function recovery after SCI via the NRF2/SLC7A11/GPX4 pathway. Reprinted with permission from S. Wu, Z. Chen, Y. Wu, Q. Shi, E. Yang, B. Zhang, Y. Qian, X. Lian, J. Xu. Copyright 2024 Elsevier [68].

The ADMSC-EXs prevent inflammation in M1 microglia and spinal cord tissues, promote M2 microglia expression, and stimulate the Nrf2/HO-1 pathway in rats with SCI [67]. ADMSC-EX can improve motor function recovery by activating the Nrf2/HO-1 pathway and microglial polarization [67]. As shown in Figure 3, ADSC-EXs also prevent ferroptosis and promote recovery of vascular and neural functions after rat SCI via the NRF2/SLC7A11/GPX4 pathway [68]. These findings highlighted the multifaceted therapeutic potential of ASC-EVs in SCI. Their ability to modulate multiple cellular processes, including neuroprotection, inflammation, and oxidative stress, suggests that ASC-EVs may offer a promising approach to repairing SCI. Additional research is needed to transform these preclinical findings into effective clinical treatments.

### 6.4. Bone Marrow Mesenchymal Stem Cell-Derived Extracellular Vesicles

The bone marrow mesenchymal stem cell-derived exosomes (BMSC-EXs) emerged as promising therapeutics for SCI due to their multifaceted effects on various cellular pathways [69]. When administered intravenously, exosomes can influence the function of macrophages in the injured spinal cord tissue by targeting M2-type macrophages [70]. In rat models of traumatic brain injury, BMSC-EXs demonstrated neuroprotective effects by reducing lesion size, improving neurobehavioral outcomes, and modulating inflammation and cell death processes [71]. The administration of BMSC-EXs enhances macrophage phagocytosis by targeting myelin debris, facilitating functional recovery post-SCI [69,72]. Moreover, they modulate A1 astrocyte activation via the nuclear factor-kappaB (NF-κB) pathway, contributing to a conducive environment for recovery in the rat SCI model [73]. The Wnt/β-catenin signaling pathway, known for its involvement in cell proliferation, neurodevelopment, and tissue repair, was also implicated in SCI recovery, as it showed great potential in promoting tissue repair and inhibiting neuronal apoptosis in rat SCI [74]. Additionally, the BCL2/Bax and Wnt/β-catenin signaling pathways play crucial roles in the pathophysiology of rat SCI, further highlighting the complexity of the molecular mechanisms involved [53].

The delivery of miRNA-133b-modified BMSC-EXs showed promise in reducing injury volume, promoting axonal regeneration, and activating key signaling molecules, including ERK1/2, STAT3, and CREB, thereby aiding in functional recovery after traumatic SCI in rat models [75]. The encapsulation of BMSC-EXs within electro-conductive hydrogels enhances their therapeutic efficacy by modulating the M2 microglial polarization via the NF-κB pathway using neural stem cell and dorsal root ganglion in vitro cell culture [76]. This modification promotes neuron and oligodendrocyte differentiation while inhibiting astrocyte differentiation, ultimately leading to increased axon outgrowth through the PTEN/PI3K/AKT/mTOR pathway in the SCI mouse model [76]. Furthermore, BMSC-EXs significantly reduce the CD68+ microglia numbers, promote neuron–axon regeneration, and improve locomotor recovery in the early stages of the mice SCI model [76].

The administration of BMSC-EVs enriched with miR-23b modulates the TLR4/NF-κB signaling pathway, reducing inflammatory processes and subsequently ameliorating SCI rats [77]. Additionally, BMSC-EXs promote miR-145-5p expression and inhibit TLR4/NF-κB pathway activation in both SCI rats and PC12 cells, suggesting their potential in preventing inflammation and related pathway activation [78]. Moreover, the sonic hedgehog (SHH) signaling pathway, which is crucial for neuronal regeneration post-injury, was targeted using BMSC-SHH-EX, which showed promising effects in promoting neuronal recovery and inhibiting astrocyte activation-related pathology after rat SCI [79]. Additionally, BMSC-EX attenuates BSCB disruption by modulating the tissue inhibitor of the metalloproteinases-2 (TIMP2)/matrix metalloproteinase (MMP) pathway, offering another avenue for therapeutic intervention in the SCI rat model [80]. Furthermore, NGF-overexpressing BMSC sheet-derived EXs repair SCI in the mouse model by facilitating neural stem cell differentiation and axonal regeneration [81]. Moreover, BMSC-EVs exhibit significant therapeutic potential in SCI by modulating multiple cellular pathways, including inflammation, neurogenesis, and axonal regeneration. These findings underscored the importance of continued research to translate the therapeutic potential of BMSC-exosomes into effective clinical treatments for SCI.

### 6.5. Dental Mesenchymal Stem Cell-Derived Extracellular Vesicles

Dental mesenchymal stem cell-derived exosomes (DSC-EXs) exhibit diverse functions, underscoring their dynamic role in intercellular communication and therapeutic potential across various pathological conditions [82]. These EXs have been implicated with many functions such as immunomodulation, neuroprotection, anti-inflammatory responses, angiogenesis, osteogenesis, and modulation of cell death in mouse SCI models [82,83].

**Figure 4:**
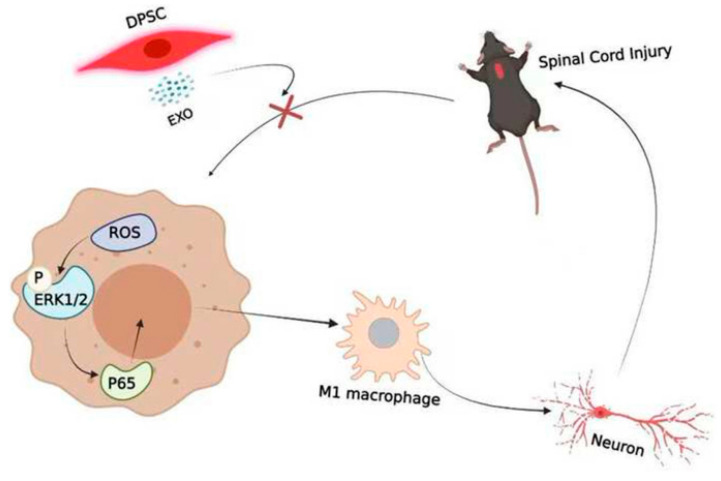
Dental pulp stem cell (DPSC)-derived exosomes can reduce macrophage M1 polarization through the ROS-MAPK-NF-κB P65 signaling pathway in SCI treatment. Disrupting the cycle between ROS and M1 macrophage polarization might also be a potentially effective treatment, as it reduces secondary damage. Reprinted with permission from Liu, C., Hu, F., Jiao, G., Guo, Y., Zhou, P., Zhang, Y., Zhang, Z., Yi, J., You, Y., Li, Z., Wang, H., Zhang, X. (2022). Copyright 2022 Journal of Nanobiotechnology, Springer Nature [84].

Figure 4 illustrates the capacity of DSC-EX to mitigate macrophage M1 polarization, particularly in the context of SCI, via the ROS-MAPK-NFκB P65 signaling pathway, suggesting their therapeutic utility in reducing secondary damage associated with the rat SCI model [84]. In the context of ischemia/reperfusion-induced cerebral injury, DSC-EX demonstrates anti-inflammatory effects, potentially mediated by the inhibition of the HMGB1/TLR4/MyD88/NF-κB pathway, highlighting their neuroprotective capabilities in mice transient middle cerebral artery occlusion injury [85]. These findings demonstrated the multifaceted therapeutic potential of DSC-EXs across various pathologies, including SCI. Their ability to modulate inflammation and protect against cellular damage suggests that DSC-EXs may offer a novel and promising approach to treating SCI. Further research is crucial to successfully translate these preclinical findings into effective clinical therapies. Table 1 demonstrate the activated signal pathways and therapeutic effects of stem cell-derived EVs in SCI.

## 7. Exploring Therapeutic Potential: Spinal Cord Tissue-Derived Extracellular Vesicles in Spinal Cord Injury

### 7.1. Neural Stem Cell-Derived Extracellular Vesicles

Many studies suggested that the transplantation of neural stem cell-derived exosomes (NSC-EXs) can enhance the motor, sensory, and autonomic nerve functions in SCI [42,89,90]. Table 2 summarizes several investigations demonstrating NSC-EX’s neuroprotective and reparative effects in SCI. These EXs were shown to protect neuronal function, mitigate neurocognitive impairment, and promote rat SCI repair [42,91]. Exosomes derived from induced pluripotent stem cell-derived neural progenitor cells exhibit neuroprotective effects against oxygen- and glucose-deprived neurons by modulating the PTEN/AKT signaling pathway and promoting neurite outgrowth in embryonic rat cortical neuron cultures [91]. Furthermore, NSC-EX-loaded with FTY720 positively affects rat SCI by regulating the PTEN/AKT pathway [92]. Notably, NSC-EXs were also found to suppress neuronal cell death by activating autophagy via the miR-374-5p/STK-4 axis in SCI mice [93]. Moreover, NSC-EXs have shown promising outcomes in reducing the size of the spinal cord cavity, enhancing microvascular regeneration, and improving Basso mouse scale scores in the SCI mice model [94]. In the SCI mice model, NSC-EX injection mitigates microglial PTEN/AKT activation and astrocyte activation while promoting the maturation of oligodendrocyte progenitor cells [95]. Additionally, NSC-EXs can facilitate spinal cord repair by modulating the cellular microenvironment of neuron cell culture in vitro study [95]. These findings highlighted the multifaceted therapeutic potential of NSC-EXs in SCI. Their ability to modulate multiple cellular processes, including neuroprotection, neurogenesis, and glial cell function, suggests that NSC-EXs may offer a promising avenue for developing novel therapies for SCI. Table 2 demonstrate the activated signal pathways and therapeutic effects of spinal cord tissue-derived EVs in SCI.

### 7.2. Schwann Cell-Derived Extracellular Vesicles

EXs released from different phenotypic Schwann cells (mature myelinating, repair, and hypothetical dysfunctional repair SCs) carry distinct cargoes with different functions [107,108]. Treatment with Schwann cell-derived exosomes (SC-EXs) reduces oxidative stress and inflammation after SCI, alleviates necroptosis, and enhances mitochondrial functionality. It also promotes mitophagy in injured PC12 cells [108]. Repair SCs secrete EXs that can enhance axonal regeneration after a nerve injury. Also, it holds significant promise for SCI repair due to the intrinsic regenerative capacity of Schwann cells in the peripheral nervous system [108]. In particular, repair SC-derived EXs contain miRNA-21 that leads to the downregulation of phosphatase and tensin homolog (PTEN) and phosphoinositide 3-kinase (PI3K) activation in the neuron cell culture [23]. Furthermore, the repair of SC-EXs increases cell viability and inhibits the apoptosis of neurons in the rat SCI model [109]. SC plays a constructive role in nerve repair and promotes axonal proliferation and de-differentiation, myelin sheath elimination, and axonal debris [110]. SC-EXs can enhance axon regeneration and mitigate inflammation through in vitro stimulation with a combination of fibroblast growth factor, neuregulin-1, and platelet-derived growth factor-AA [111]. It can also contribute to axon protection by enhancing autophagy and anti-apoptotic effects via the EGFR/Akt/mTOR signaling pathway in the rat SCI model.

Contrarily, SC-EX can stimulate the expression of TLR2 in astrocytes after SCI and reduce the deposition of CSPGs through NF-κB/PI3K signaling, thereby promoting functional recovery after SCI in mice [101]. Ren et al. revealed that SC-EX with MFG-E8 modifies macrophage/microglial polarization to reduce inflammation and enhance motor function via the SOCS3/STAT3 pathway after SCI contusion in a rat model [103]. The skin precursor SC-EXs triggered axonal outgrowth and the regeneration of motor and sensory neurons through the PI3K/protein kinase B/mammalian target of rapamycin/ribosomal protein S6kinase β-1 (PI3K/Akt/mTOR/p70S6K) signaling pathway in peripheral nerve injury (PNI) and SCI in rat dorsal root ganglion and sensory neurons culture in vitro [112]. SC-EXs also showed a neuroprotective effect on neurons by blocking the caspase-3 cell death pathway, consistent with axonal regeneration and promoting cell survival in motor neurons in vitro study [113]. The skin precursor-derived SC-EVs enhanced neuron growth and survival, particularly in oxygen-glucose-deprivation-injured rat sensory neurons from dorsal root ganglion, through miR-21-5p’s regulation of the PTEN/PI3K/Akt pathway [112]. SC-EVs demonstrate significant therapeutic potential in SCI. By modulating key signaling pathways and influencing cellular processes such as inflammation and neurogenesis, SC-EVs contribute to improved functional recovery after SCI.

### 7.3. Macrophage-Derived Extracellular Vesicles

The peripheral macrophages efficiently promote anti-inflammation in injured areas and are the significant factors that support healing after in vitro study of anti-inflammatory microglial polarization following autophagy induction [99]. Furthermore, it highlights the potential of macrophage-derived exosomes (MP-EXs) in modulating the anti-inflammatory process by stimulating microglial autophagy via the inhibition of the PI3K/AKT/mTOR signaling pathway in rat SCI [99]. MP-EXs-loaded baicalin was found to improve solubility and brain targeting ability, leading to significant neuroprotection in ischemic stroke patients through the antioxidative pathway [114]. In another study, OTULIN derived from macrophage 2-derived EXs emerges as a crucial mediator in facilitating angiogenic effects by directly deubiquitinating β-catenin and inducing the expression of genes associated with angiogenesis in spinal cord microvascular endothelial cells after mouse SCI [98].

In the SCI mice model, M2-derived EXs-loaded berberine had anti-inflammatory and anti-apoptotic effects by repolarizing macrophages from the M1 to M2 phenotype [115]. In particular, EXs M2 macrophage alleviated tissue damage, enhanced functional recovery, and increased angiogenesis after the SCI rat model [116]. Also, it can improve tube migration, proliferation, and formation and partially activate the HIF-1/VEGF signaling pathway through in vitro study of brain endothelial cell lines [116]. M2 macrophage-derived EXs may promote M2 macrophage polarization via the miRNA–mRNA network, making them a promising therapeutic agent for SCI immune microenvironment in vitro. The miR-23a-3p/PTEN/PI3K/AKT axis is notable in the network shown in the SCI rat model [100]. In conclusion, MP-EXs, particularly those derived from M2 macrophages, exhibit significant therapeutic potential in SCI. They can modulate the inflammatory response by promoting anti-inflammatory macrophage polarization, inhibiting inflammation, and enhancing angiogenesis. These findings suggested that MP-EVs may offer a promising approach for improving functional recovery after SCI.

### 7.4. Microglia-Derived Extracellular Vesicles

Microglia, a type of neuroglia analogous to macrophages in the brain and spinal cord, are recognized as pivotal contributors to the pathophysiology of mice SCI [117]. The miR-151-3p is highly expressed in microglia-derived exosomes (MG-EXs), and thus, it exerts a neuroprotective effect during SCI repair [72]. Concurrently, MG-EXs can activate the p53/p21/CDK1 signaling cascade, thereby regulating neuronal apoptosis and promoting axonal growth after contusive mouse SCI [72]. Additionally, MG-EXs may function as an antioxidant by activating the Keap1/Nrf2/HO-1 pathway, thereby facilitating functional recovery in the SCI mouse model [97]. Furthermore, MG-EXs miR-124–3p can potentially mitigate neuronal degeneration and improve cerebral outcomes via the Rela/ApoE signaling pathway [118]. Moreover, cell polarization shifts from the M1 to M2 phenotype may occur in various subsets of microglial and monocyte cells after SCI in mice. M2 MG-EXs enhance neuron survival and protection, promote motor function recovery, and reduce the lesion area size [119]. Lastly, neuroprotection is influenced by reduced A1 astrocyte activation by inhibiting NF-κB signaling pathway activation in mice SCI [119]. Overall, MG-EVs play a crucial role in the pathophysiology of SCI. They can exert neuroprotective effects by regulating neuronal apoptosis, activating antioxidant pathways, and modulating microglial polarization. These findings suggest that MG-EVs may offer a promising therapeutic target for improving functional outcomes after SCI.

### 7.5. Astrocyte-Derived Extracellular Vesicles

Astrocytes can play a pivotal role in the nervous system, such as neuron nourishment, promoting synapse construction, synaptic pruning via phagocytosis, regulation of cerebral blood flow, and homeostasis maintenance [120]. A1 and A2 reactive astrocytes are distinctive in acute neurotic conditions after mouse cortex stab injury [121]. A1 astrocytes are strongly neurotoxic, whereas A2 astrocytes might deal with neuro-protective effects by increasing the neurotrophic factors by microglial cells in vitro [122]. The astrocyte-derived exosomes (AC-EXs) treated with semaphorin 3A-I enhance the axonal outgrowth and PTGDS expression via neuronal Rnd1/R-Ras/Akt/glycogen synthase kinase 3β (GSK-3β) signaling and promote axon regeneration and stroke recovery in traumatic brain injury (TBI) in rats [123]. In the SCI rat model, AC-EVs facilitate tissue repair, decrease fibrosis, and enhance limb function and walking ability [124]. EVs derived from LPS-stimulated astrocytes (LPS-AS-EVs) exhibited enhanced neurite outgrowth in vitro and improved motor function recovery in vivo compared to EVs from unstimulated astrocytes (AS-EVs) in an SCI rat model [106]. Mechanistically, both AS-EVs and LPS-AS-EVs modulated the Hippo pathway in PC12 cells by increasing monopole spindle binding protein 1(MOB1) and decreasing Yes-associated protein (YAP), thereby promoting neurite elongation [106]. This finding suggested that LPS-stimulated astrocytes release EVs that enhance neuronal repair through the MOB1–YAP axis, providing a potential therapeutic avenue for SCI treatment [106]. While studies explored the therapeutic potential of AC-EVs in TBI, their application in SCI remains limited. However, given the similarities in injury mechanisms and treatment approaches between TBI and SCI, both of which are central nervous system injuries, we anticipate a surge in research investigating AC-EVs-mediated repair of SCI. We believe that AC-EVs hold significant promise as therapeutic agents for SCI.

### 7.6. Pericyte-Derived Extracellular Vesicles

Pericyte-derived exosomes (PC-EXs) can protect the endothelial cells and endothelial BSCB in hypoxic conditions via the PTEN/AKT pathway after mice SCI [104]. Notably, PC-EXs can increase the blood flow and oxygen supply to the damaged area and improve motor function after SCI [104]. Pericyte proliferation may occur through the S1P/S1PR3 pathway, leading to significantly reduced glial scar formation and improved locomotor recovery after SCI in rats [125]. In addition, S1P/S1PR3 signaling promoted pericyte proliferation via the Ras/pERK pathway, as shown by the reduced pericyte proliferation with CAY10444, an S1PR3 inhibitor. [125]. Thus, PC-EVs demonstrated the significant therapeutic potential in SCI by promoting endothelial function, enhancing blood flow, and stimulating pericyte proliferation, ultimately leading to reduced glial scarring and improved functional recovery.

## 8. Therapeutic Potential of Extracellular Vesicles from Other Cell Types in Spinal Cord Injury

The activation of the JAK2/STAT3 pathway by miR-214-containing exosomes derived from muscle-derived stem cells may facilitate the regeneration and repair of peripheral neurons following a rat sciatic nerve crush injury, potentially through the inhibition of PTEN [126]. T cell-derived exosomes can modulate immune responses, as evidenced by the enrichment of RAS signaling pathway proteins in exosomes from activated T cells, leading to ERK phosphorylation [127]. Gingiva-derived MSC-EXs successfully regenerate the sciatic nerve in rodent models [128]. A recent study found that EXs derived from fibroblasts can promote retinal ganglion cell neurite growth by activating mTOR and promoting Wnt10b in neuron cell culture in vitro [129]. These studies play a role in axonal regrowth and regenerative signaling [129]. Additionally, human menstrual blood-derived mesenchymal stem cell-derived exosomes demonstrated the potential to promote axon regeneration in a rat model of SCI [130]. EXs from human urine stem cells show promise for regeneration, aiding spinal cord recovery by fostering angiogenesis through Angiopoietin-like 3 transfer in the contusion SCI mouse model [131]. EXs derived from Treg cells can encapsulate and transport miR-2861, thereby modulating the expression of IRAK1 to influence BSCB integrity and motor function following SCI in mice [132]. Pericyte-derived exosomes containing miR-210-5p were shown to enhance endothelial barrier function by inhibiting JAK1/STAT3 signaling. This mechanism, linked to reduced lipid peroxidation and improved mitochondrial function, offers a promising therapeutic target for restoring the BSCB after SCI [133]. Locally administered gingival mesenchymal stem cell-derived EVs enhanced nerve regeneration and functional recovery in injured mice by upregulating c-JUN, a key gene for Schwann cell repair [134]. Endothelial progenitor cell-derived exosomes containing miR-222-3p modulated macrophage polarization via the SOCS3/JAK2/STAT3 pathway, improving functional recovery in mice with SCI [135]. Hence, EVs derived from diverse cellular sources exhibit potent regenerative properties, influencing key cellular processes, including immune modulation, angiogenesis, and the regulation of crucial signaling pathways. These findings underscored the significant therapeutic potential of EVs for SCI.

## 9. Exploring the Role of Bioinformatics in Advancing Stem Cell-Derived Extracellular Vesicle Studies

Bioinformatics (BI) is a scientific subdiscipline using computer technology to collect, store, analyze, and disseminate biological information [136]. Post-SCI, BI analysis can help identify the hub ferroptosis genes, including STAT3, JUN, TLR4, ATF3, HMOX1, MAPK1, MAPK9, PTGS2, VEGFA, and RELA, and the potential drugs targeting ferroptosis to repair SCI in rats [137]. The integrated BI analysis, including differential expression gene, gene ontology enrichment, KEGG pathway analysis, and protein–protein interaction network, helped to reveal that the Sema3A/NRP1 signaling may regulate the development of oligodendrocytes post-SCI, thereby affecting functional recovery [138]. For SCI-induced neuropathic pain in a murine model [139], 592 differentially expressed genes with a significantly altered expression were found, with CCl3 showing the highest upregulation, and 209 pathways changed significantly based on the results of the KEGG analysis with MAPK signaling pathway [140]. Studies showed significant alterations in the expression of ferroptosis-related genes in spinal cord tissue following SCI. For example, Dong et al. identified 48, 44, and 27 differentially expressed ferroptosis genes at 1, 3, and 7 days post-injury, respectively [141]. Subsequent research consistently observed these changes in ferroptosis-related gene expression within the injured spinal cord [142,143]. Ferroptosis, distinct from apoptosis, involves iron-dependent lipid peroxidation [144]. After SCI, the breakdown of red blood cells leads to localized iron overload and increased reactive oxygen species, all contributing to ferroptosis [145]. Yao et al. noted that deferoxamine can inhibit gliosis and enhance neuron survival by blocking this process [146]. These findings highlight the crucial role of ferroptosis in the pathophysiology of SCI and suggest that targeting this pathway may offer novel therapeutic avenues for improving functional recovery.

### Unveiling Therapeutic Pathways via microRNA Analysis in Extracellular Vesicles Transplanted Rodents

miRNAs can regulate cell differentiation, proliferation, and apoptosis and are closely linked with pathological processes (inflammation, demyelination, oxidative stress, and neural apoptosis) after SCI [74]. Engineered non-vesicular nanoparticles offer a complementary approach for delivering bioactive molecules, including miRNAs [147]. These precisely tailored particles overcome the limitations of EVs by enabling the encapsulation of specific cargo, enhancing stability in circulation and at the target site, and facilitating targeted delivery to desired cell types through functionalization [147]. Although EXs are known to transfer various bioactive molecules, including microRNAs and proteins, to recipient cells, the associated mechanism of exosome-mediated SCI repair still needs further exploration [148,149]. Thus, RNA sequencing was performed to identify the key pathways, and gene analyses can help to differentiate groups. Li et al. revealed improved survival of neurons and motor function after the systemic injection of miRNA-133b EX, which moderately activates the CREB, STAT3, ERK1/2, and RhoA signaling in rat SCI [12]. Also, MSC-EXs miRNA-133b considerably promotes the expression of GFAP, MBP, NF, and GAP43 expression, prompting axon regeneration and improving functional recovery in SCI animals [150]. Furthermore, it can deliver miR-133b to increase neurite growth and stimulate neuronal plasticity and functional recovery [151]. The umbilical MSC-EXs miR-199a-3p/145–5p can help in the functional recovery of the SCI in a rat model via the mediated NGF/TrkA signaling pathway [54]. Weihua et al. recommended that miR-216a-5p from MSC-EXs involves microglial polarization [6]. Hung et al. suggested that miR-21 plays a significant role in nerve regeneration and protection in stroke [152]. Emerging evidence indicates that exosomal miR-423-5p, originating from osteoblasts activated by cerebrospinal fluid pulsation stress, plays a pivotal role in enhancing endothelial cell angiogenesis through the modulation of the DUSP8/ERK1/2 signaling pathway [153]. Table 3 shows the signal pathways and therapeutic effects of EVs miRNAs in SCI. 

The miRNAs regulate the expression of related proteins by upregulating or downregulating target genes that are altered after SCI. For example, the changes in miR-10a, miR-10b, miR-142-3p, miR-338, and miR-133 contents after SCI are closely related to the pathogenesis of the disease [158]. BmMSC-EXs inhibit the NF-κB pathway by upregulating miR-23b targeting TLR4, participating in the process of oxidative stress, alleviating the inflammatory response after SCI, and improving the motor function of rats after SCI [77]. miR-29a/199B inhibits the RGMA/STAT3 axis and promotes neural function repair in rats post-SCI [159]. NSC-EXs regulate astrocyte and microglia activation through miR-124-3p to protect against traumatic SCI [160]. The overexpression of miR-223 decreased the protein expression levels of interleukin (IL)-1β, IL-18, NLRP3, ASC, and caspase-1, and regulated the transformation of macrophages between types in injured spinal cords of mice with chronic sciatic nerve injury [161]. Additionally, miR-20a, miR-21, miR-497, miR-494, miR-223, miR-29b, miR-320, and miR-124 were involved in cell apoptosis after SCI [162,163,164,165]. Furthermore, miR-133b, miR-20a, and miR-124 were involved in promoting angiogenesis and regulating nerve repair after SCI [166,167,168]. MSCs-EXs can reduce cell proliferation and neuronal ferroptosis caused by hypoxia [169]. This is achieved by inhibiting neuronal cell ferroptosis through the miR-5627-5p/FSP1 axis, thereby reducing neuronal dysfunction [169].

Exosomal miR-124-3p from neurons can reduce the activation of M1 microglia in mice after SCI via PI3K/AKT/NF-κB signaling [160]. Huang et al. discovered that exosomal miR-494 prevented inflammation and neuron apoptosis in the injured area, supporting nerve generation and motor function recovery in rats with SCI [163]. This review highlighted the critical role of miRNAs within EVs in modulating various cellular processes involved in SCI repair. miRNAs encapsulated within EVs derived from multiple sources, including mesenchymal stem cells, neural stem cells, and macrophages, regulate key signaling pathways such as NF-κB, PI3K/AKT, and ERK1/2, influencing inflammation, neurogenesis, angiogenesis, and cell survival. These findings underscored the therapeutic potential of utilizing and manipulating exosomal miRNAs to enhance functional recovery after SCI. Further research is crucial to elucidate the precise mechanisms of miRNA action within the injured spinal cord and to translate these findings into effective clinical therapies for SCI.

## 10. Clinical Studies

Despite limitations in search terms and incomplete data, the surge in EV-related clinical trials (471 since 1999) reflects the growing interest in their therapeutic potential. One promising example is NCT03675885, which evaluates the safety and efficacy of stem cell-derived exosomes for SCI. This trial’s initial results demonstrate some patients’ sensory improvements and motor recovery, highlighting the potential for clinical translation [170]. While we anticipate no change in our conclusions, it may be necessary to repeat this analysis later to identify trends in these trials. A further limitation is that our search terms were not exhaustive, focusing only on “extracellular vesicles” and “exosomes”, the most common terms for small EVs. However, other relevant subcategories exist, such as oncosomes, microsomes, and ectosomes, which could also be explored in future studies. Finally, clinicaltrials.gov lacks strict reporting requirements, resulting in many vague and incomplete study records. This analysis serves as a preliminary assessment of the current landscape of EV clinical trials to guide future research in SCI.

## 11. Navigating Future Insights and Anticipated Challenges

### 11.1. Future Insights

1. Enhanced targeting and delivery: Advancements in engineering will enable precise targeting of stem cell exosomes to injured spinal cord regions and specific cell types, maximizing therapeutic efficacy [171].
2. Synergistic Therapies: Integrating EVs with biomaterials, growth factors, and other therapies can significantly improve tissue repair and functional recovery [74].
3. Personalized Medicine: Tailoring EV therapies to individual patients, utilizing omics technologies to select optimal donors and optimize exosome composition, can improve treatment outcomes [172].
4. Non-invasive Monitoring: Implementing non-invasive imaging and biomarker assays will provide valuable insights into exosome biodistribution, persistence, and therapeutic response, guiding treatment optimization.

### 11.2. Challenges

Establishing standardized protocols for EV isolation, purification, and characterization is imperative to ensure the reproducibility and comparability of preclinical and clinical studies [74].

## 12. Conclusions

MSC-derived EVs represent a promising therapeutic avenue for SCI due to their multifaceted mechanisms of action and unique ability to traverse biological barriers. Existing preclinical research suggests the potential benefits of MSC-EVs in promoting tissue repair, attenuating inflammation, and enhancing functional recovery in SCI models. However, further investigation is warranted to fully understand their therapeutic potential. Nevertheless, the growing body of evidence supporting the therapeutic potential of MSC-EVs offers hope for developing novel and effective treatments for SCI.

## Figures and Tables

**Table 1:** Comparative therapeutic efficacy of different cell types and their extracellular vesicles in animal models of spinal cord injury.

Cell Type/EV Type	Animal Model	Delivery Route	Signaling Pathways	Functions	Reference
HucMSC-EXs	Rat chronic constriction injury	Intrathecal injection	TLR2/MyD88/NF-κB	Attenuates neuropathic pain	[56]
HucMSC-EXs	Rat spinal cord injury	Tail vein injection	Wnt/β-catenin	Mitigates apoptosis downregulates inflammatory factors and promotes angiogenesis and axonal growth	[53,54]
HucMSC-EXs	Rat spinal cord injury	Intravenous administration	BCL2/Bax and Wnt/β-catenin	Productive effects of SCI	[53]
HucMSC-EXs	LPS-treated mouse model	Tail vein injection	NRF2/NF-κB/NLRP3	Reduces oxidative stress and neuroinflammation	[59]
HPMSC-EXs	Rat spinal cord injury	Tail vein injection	MEK/ERK/cAMP-CREB	Endogenous neurogenesis activation enhances recovery from spinal cord injury	[60]
ADSC-EXs	Rat spinal cord injury	Tail vein injection	Nrf2/HO-1	Prevents inflammation in M1 microglia and spinal cord tissues	[67]
ADSC-EXs	Rat spinal cord injury	Tail vein injection	NRF2/SLC7A11/GPX4	Prevents ferroptosis and promotes recovery of vascular and neural functions	[68]
ADMSC -EXs	Mice spinal cord injury	Tail vein injection	NF-κB light-chain enhancer (related to STAT activity) to inhibit NF-κB	Repairs SCI via Shifting microglial M1/M2 Polarization	[86]
BmMSC-EXs	Rat spinal cord injury	Tail vein injection	Wnt/β-catenin	Inhibits neuronal apoptosis and promotes motor function recovery	[87]
BmMSC-EXs	Rat spinal cord injury	Intravenous administration	Wnt/β-catenin	Plays crucial roles in SCI	[53]
Bone MSC-EXs	Mice spinal cord injury	Tail vein injection	TLR4–NF-κB and activating the PI3K–AKT	Exosome-shuttled miR-216a-5p from hypoxic pre-conditioned MSC repairs traumatic SCI	[6]
BMSC-SHH-EXs	Rat spinal cord injury	Intravenous injection	Sonic hedgehog (SHH)	Promotes neuron recovery and inhibits astrocyte activation	[79]
BmMSC-EXs	Rat acute spinal cord injury	Intravenous injection	TIMP2)/MMP	Therapeutics intervention in acute SCI	[80]
BmMSC-EXs	Rat diabetic nephropathy model	Tail vein injection	JAK2/STAT3	Protective effects of diabetic nephropathy and its possible mechanism	[88]
miR-216a-5p was enriched in MSC-derived exosomes	Rat spinal cord injury	Tail vein injection	TLR4/NF-κB/PI3K/AKT	Repairs traumatic SCI by suppressing the activation of A1 neurotoxic reactive astrocytes	[75]
DSC-EXs	Rat spinal cord injury	Tail vein injection	ROS-MAPK-NF-κB P65	Suppresses M1 macrophage polarization	[84]
DSC-EXs	Mice transient middle cerebral artery occlusion injury	Tail vein injection	HMGB1/TLR4/MyD88/NF-κB	Promotes anti-inflammatory and neuroprotective effects	[85]

HucMSC-EXs: Human umbilical cord mesenchymal stem cell-derived exosome; BmMSC-EXs: Bone marrow mesenchymal stem cell-derived exosome; ADMSC-EXs: Adipose stem cell-derived exosome; DSC-EXs: Dental stem cell-derived exosome.

**Table 2:** Cellular signaling pathways are activated by spinal cord tissue-derived extracellular vesicles in spinal cord injury.

Cell Type/EV Type	Injury Model	Delivery Route	Signal Pathways	Therapeutic Effects	Reference
MG-EXs	Mice spinal cord contusive injury	Exogenous administration	p53/p21/CDK1	Regulates neuronal apoptosis and promotes axonal growth	[96]
MG-EXs	Mouse spinal cord injury	Tail vein injection	Keap1/Nrf2/HO-1	Promotes functional recovery after SCI	[97]
MP-EXs	Mouse spinal cord injury	Locally administrated at the injury site	Wnt/β-Catenin	Promotes angiogenesis after SCI.	[98]
MP-EXs	Rat spinal cord contusion injury	Tail vein injection	PI3K/AKT/mTOR	Attenuates anti-apoptosis suppresses BSCB disruption and functional recovery after SCI.	[99]
MP-EXs	Rat spinal cord injury	Tail vein injection	miR-23a-3p/PTEN/PI3K/AKT axis	Phenotypic switch of macrophages in the immune microenvironment	[100]
SC-EXs	Mice spinal cord contusion injury	Tail vein injection	NF-κB/PI3K	Stimulates the expression of TLR2 in astrocytes after SCI and reduces the deposition of CSPGs.	[101]
SC-EXs	Rat spinal cord injury	Tail vein injection	vincristine receptor B	Reduces apoptosis and promotes recovery of motor function	[102]
SC-EXs	Rat spinal cord contusion model	Tail vein injection	SOCS3/STAT3	Attenuates inflammation	[103]
PC-EXs	Mice spinal cord injury	Tail vein injection	PTEN/Akt	Improves endothelial barrier function under hypoxic conditions and protects endothelial cells	[104]
NSC-EXs	Rat spinal cord injury	Tail vein injection	PTEN/AKT	Promotes functional recovery of SCI	[92]
NSC-EXs	Rat acute spinal cord injury	Tail vein injection	miR-219a-2-3p/YY1	Inhibits neuro-inflammation and promotes neuroprotection	[105]
AC-EVs	Spinal cord injury	In vitro PC12 cell culture	Hippo pathwayMOB1-YAP axis	Promotes neurite elongation	[106]

MG-EX: Microglial cell-derived exosome; MP-EXs: Macrophage cell-derived exosome; SC-EXs: Schwann cell-derived exosomes; PC-EXs: Pericyte cell-derived exosome; NSC-EXs: Neuron stem cell-derived exosome; AC-EXs: Astrocyte cell-derived exosome; SCI: Spinal cord injury; BSCB: Blood–spinal cord barrier.

**Table 3:** Possible signaling pathways involved in different types of microRNA from mesenchymal stem cell (MSC)-derived extracellular vesicles.

Cell Type/EV Type	Exosome Cargo	Delivery Route	Injury Model	Signaling Pathways	Functions	Reference
HucMSC-EXs	miR-145-5p	Tail vein injection	Rat spinal cord injury	TLR4/NF-κB	Regulates inflammation	[78]
HucMSC-EXs	miR-199a-3p/145-5p	Tail vein injection	Rat spinal cord injury	NGF/TrkA	Promotes neuroprotective and functional recovery	[54]
MP-EXs	miR-155	Tail injection	Mouse contusive spinal cord injury	NF-κB; miR-155/SOCS6/p65 axis	Ensures the transport network between macrophages and vascular endothelial cells after SCI	[154]
MSC-EXs	miR-338-5p	Tail vein injection	Rat spinal cord injury	Cnr1/Rap1/Akt	Reduces apoptosis and promotes neuronal survival	[155]
ADSC-EXs	miR-133b	Tail intravenous injection	Rat spinal cord injury	-	Promotes axonal regrowth and motor function recovery	[12]
BmMSC-EXs	miR-23b	Caudal vein injection	Rat spinal cord injury	targeting TLR4 and inhibiting NF-κB pathway activation	Alleviates spinal cord injury	[77]
BmMSC-EXs	miR-544	Intravenous injection	Rat spinal cord injury	-	Reduces the number of apoptotic neurons	[156]
BmMSC-EXs	miR-125a	Intravenous injection	Rat spinal cord injury	-	Promotes M2 macrophage polarization	[157]

HucMSC-EXs: Human umbilical cord mesenchymal stem cell-derived exosome; BmMSC-EXs: Bone marrow mesenchymal stem cell-derived exosome; ADSC-EXs: Adipose stem cell-derived exosome; SCI: Spinal cord injury.

## Data Availability

Not applicable.

## Abbreviations

MSC-EVs	Mesenchymal stem cell-derived extracellular vesicles
MSC-EXs	Mesenchymal stem cell-derived exosomes
SCI	Spinal cord injury
miRNAs	Micro RNAs
BSCB	Blood–spinal cord barrier
HucMSC-EXs	Human umbilical cord mesenchymal stem cell-derived exosomes
hPMSC-EXs	Human placental mesenchymal stem cell-derived exosomes
ADSC-EXs	Adipose mesenchymal stem cell-derived exosomes
BMSC-EXs	Bone marrow mesenchymal stem cell-derived exosomes
DSC-EXs	Dental mesenchymal stem cell-derived exosomes
NSC-EXs	Neural stem cell-derived exosomes
SC-EXs	Schwann cell-derived exosomes
MP-EXs	Macrophage-derived exosomes
MG-EXs	Microglia-derived exosomes
AC-EXs	Astrocyte-derived exosomes
PC-EXs	Pericyte-derived exosomes
TLR2	Toll-like receptor 2
NF-κB	Nuclear factor kappa-light-chain-enhancer of activated B cells
Rsad2	Radical SAM domain-containing 2
BCL2	B-cell lymphoma 2
Bax	Bcl-2-associated protein x
TrkA	Tropomyosin receptor kinase A
NGF	Nerve growth factor
IRAK1	Interleukin-1 receptor-associated kinases 1
TRAF6	TNF receptor-associated factor 6
NRF2	Nuclear factor erythroid 2-related factor 2
NLRP3	Nucleotide-binding domain, leucine-rich-containing family, pyrin domain–containing-3
NPCs	Neural pluripotent cells
MEK	Mitogen-activated protein kinase
ERK	Extracellular signal-regulated kinase
CREB	cAMP-response element binding protein
OGD/R	Oxygen-glucose deprivation/reperfusion
Nrf2/HO-1	Nuclear factor erythroid-2 related factor 2/heme oxygenase
PI3K/Akt	Phosphatidylinositol 3-kinase/Protein kinase B
SLC7A11	Solute carrier family 7 member 11
GPx4	Glutathione peroxidase 4
PTGDS	Prostaglandin D2 synthase
Rnd1	Rho Family GTPase 1
R-Ras	R-Ras gene
S1P	Sphingosine-1-phosphate
SIPR3	Sphingosine-1-phosphate receptor 3
Sema3A	Semaphorin 3A
NRP1	Neuropilin 1
SOCS6	Suppressor of cytokine signaling 6
Rap1	Repressor/Activator Protein 1
Cnr1	Cannabinoid receptor gene
KEGG	Kyoto Encyclopedia of Genes and Genomes
STAT3	Signal transducer and activator of transcription 3
JUN	Jun proto-oncogene or enhancer-binding protein
HMOX1	Heme oxygenase 1
PTGS2	Prostaglandin-Endoperoxide Synthase 2
VEGFA	Vascular endothelial growth factor A
RELA	v-rel avian reticuloendotheliosis viral oncogene homolog A
CCl3	C-C Motif Chemokine Ligand 3
PTEN	Phosphatase and tensin homolog
SHH	Sonic hedgehog
TIMP2	Tissue inhibitor of metalloproteinases-2
MMP	Matrix metalloproteinase
ROS	Reactive oxygen species
MAPKs	Mitogen-activated protein kinases
Cdc42	Cell division control protein 42 homolog
HMGB1	High mobility group box 1
EGFR	Epidermal growth factor receptor
mTOR	Mammalian target of rapamycin
PKB also known as AKT	Protein kinase B
PI3Ks	Phosphoinositide 3-kinases
DUSPs	dual-specificity phosphatases

## References

[1] Poongodi R., Yang T.-H., Huang Y.-H., Yang K.D., Chen H.-Z., Chu T.-Y., Wang T.-Y., Lin H.-C., Cheng J.-K. (2024). Stem cell exosome-loaded Gelfoam improves locomotor dysfunction and neuropathic pain in a rat model of spinal cord injury. Stem Cell Res. Ther..

[2] Hu X., Xu W., Ren Y., Wang Z., He X., Huang R., Ma B., Zhao J., Zhu R., Cheng L. (2023). Spinal cord injury: Molecular mechanisms and therapeutic interventions. Signal Transduct. Target. Ther..

[3] Yan L., Fu J., Dong X., Chen B., Hong H., Cui Z. (2022). Identification of hub genes in the subacute spinal cord injury in rats. BMC Neurosci..

[4] Shen P.-P., Wang B., Li Y. (2021). Induced pluripotent stem cell technology for spinal cord injury: A promising alternative therapy. Neural Regen. Res..

[5] Tran A.P., Warren P.M., Silver J. (2021). New insights into glial scar formation after spinal cord injury. Cell Tissue Res..

[6] Liu W., Rong Y., Wang J., Zhou Z., Ge X., Ji C., Jiang D., Gong F., Li L., Chen J. (2020). Exosome-shuttled miR-216a-5p from hypoxic preconditioned mesenchymal stem cells repair traumatic spinal cord injury by shifting microglial M1/M2 polarization. J. Neuroinflammation.

[7] Khalatbary A.R. (2021). Stem cell-derived exosomes as a cell free therapy against spinal cord injury. Tissue Cell.

[8] Zhao C., Zhou X., Qiu J., Xin D., Li T., Chu X., Yuan H., Wang H., Wang Z., Wang D. (2019). Exosomes Derived from Bone Marrow Mesenchymal Stem Cells Inhibit Complement Activation in Rats With Spinal Cord Injury. Drug Des. Dev. Ther..

[9] Ren Z., Qi Y., Sun S., Tao Y., Shi R. (2020). Mesenchymal Stem Cell-Derived Exosomes: Hope for Spinal Cord Injury Repair. Stem Cells Dev..

[10] Lai R.C., Yeo R.W.Y., Tan K.H., Lim S.K. (2013). Exosomes for drug delivery—A novel application for the mesenchymal stem cell. Biotechnol. Adv..

[11] Huang J.-H., Xu Y., Yin X.-M., Lin F.-Y. (2020). Exosomes Derived from miR-126-modified MSCs Promote Angiogenesis and Neurogenesis and Attenuate Apoptosis after Spinal Cord Injury in Rats. Neuroscience.

[12] Li D., Zhang P., Yao X., Li H., Shen H., Li X., Wu J., Lu X. (2018). Exosomes Derived From miR-133b-Modified Mesenchymal Stem Cells Promote Recovery After Spinal Cord Injury. Front. Neurosci..

[13] Tang H., Li J., Wang H., Ren J., Ding H., Shang J., Wang M., Wei Z., Feng S. (2023). Human umbilical cord mesenchymal stem cell-derived exosomes loaded into a composite conduit promote functional recovery after peripheral nerve injury in rats. Neural Regen. Res..

[14] Liu J.-S., Du J., Cheng X., Zhang X.-Z., Li Y., Chen X.-L. (2019). Exosomal miR-451 from human umbilical cord mesenchymal stem cells attenuates burn-induced acute lung injury. J. Chin. Med. Assoc..

[15] Morita T., Sasaki M., Kataoka-Sasaki Y., Nakazaki M., Nagahama H., Oka S., Oshigiri T., Takebayashi T., Yamashita T., Kocsis J.D. (2016). Intravenous infusion of mesenchymal stem cells promotes functional recovery in a model of chronic spinal cord injury. Neuroscience.

[16] Lv L., Sheng C., Zhou Y. (2019). Extracellular vesicles as a novel therapeutic tool for cell-free regenerative medicine in oral rehabilitation. J. Oral Rehabil..

[17] Kiyotake E.A., Martin M.D., Detamore M.S. (2020). Regenerative rehabilitation with conductive biomaterials for spinal cord injury. Acta Biomater..

[18] El Andaloussi S., Mäger I., Breakefield X.O., Wood M.J.A. (2013). Extracellular vesicles: Biology and emerging therapeutic opportunities. Nat. Rev. Drug Discov..

[19] He C., Zheng S., Luo Y., Wang B. (2018). Exosome Theranostics: Biology and Translational Medicine. Theranostics.

[20] Liu W.-Z., Ma Z.-J., Li J.-R., Kang X.-W. (2021). Mesenchymal stem cell-derived exosomes: Therapeutic opportunities and challenges for spinal cord injury. Stem Cell Res. Ther..

[21] Wu S.-C., Kuo P.-J., Rau C.-S., Wu Y.-C., Wu C.-J., Lu T.-H., Lin C.-W., Tsai C.-W., Hsieh C.-H. (2021). Subpopulations of exosomes purified via different exosomal markers carry different microRNA contents. Int. J. Med. Sci..

[22] Hade M.D., Suire C.N., Suo Z. (2021). Mesenchymal Stem Cell-Derived Exosomes: Applications in Regenerative Medicine. Cells.

[23] López-Leal R., Díaz-Viraqué F., Catalán R.J., Saquel C., Enright A., Iraola G., Court F.A. (2020). Schwann cell reprogramming into repair cells increases miRNA-21 expression in exosomes promoting axonal growth. J. Cell Sci..

[24] Fonseka P., Mathivanan S. (2023). Extracellular Vesicles Biogenesis, Cargo Sorting and Implications in Disease Conditions. Cells.

[25] Karnas E., Dudek P., Zuba-Surma E.K. (2023). Stem cell- derived extracellular vesicles as new tools in regenerative medicine—Immunomodulatory role and future perspectives. Front. Immunol..

[26] Amin S., Massoumi H., Tewari D., Roy A., Chaudhuri M., Jazayerli C., Krishan A., Singh M., Soleimani M., Karaca E.E. (2024). Cell Type-Specific Extracellular Vesicles and Their Impact on Health and Disease. Int. J. Mol. Sci..

[27] Newman L.A., Fahmy A., Sorich M.J., Best O.G., Rowland A., Useckaite Z. (2021). Importance of between and within Subject Variability in Extracellular Vesicle Abundance and Cargo when Performing Biomarker Analyses. Cells.

[28] Zanirati G., dos Santos P.G., Alcará A.M., Bruzzo F., Ghilardi I.M., Wietholter V., Xavier F.A.C., Gonçalves J.I.B., Marinowic D., Shetty A.K. (2024). Extracellular Vesicles: The Next Generation of Biomarkers and Treatment for Central Nervous System Diseases. Int. J. Mol. Sci..

[29] Yao X., Liu Z., Huang Z., Liu J., Sun B., Zhu Q., Ding R., Chen J. (2021). Proteomics and bioinformatics reveal insights into neuroinflammation in the acute to subacute phases in rat models of spinal cord contusion injury. FASEB J..

[30] Cheng X., Zheng Y., Bu P., Qi X., Fan C., Li F., Kim D.H., Cao Q. (2018). Apolipoprotein E as a novel therapeutic neuroprotection target after traumatic spinal cord injury. Exp. Neurol..

[31] Li Y., Luo W., Meng C., Shi K., Gu R., Cui S. (2024). Exosomes as promising bioactive materials in the treatment of spinal cord injury. Stem Cell Res. Ther..

[32] Shen Y., Cai J. (2022). The Importance of Using Exosome-Loaded miRNA for the Treatment of Spinal Cord Injury. Mol. Neurobiol..

[33] Yang X.-X., Sun C., Wang L., Guo X.-L. (2019). New insight into isolation, identification techniques and medical applications of exosomes. J. Control. Release.

[34] Akbar A., Malekian F., Baghban N., Kodam S.P., Ullah M. (2022). Methodologies to Isolate and Purify Clinical Grade Extracellular Vesicles for Medical Applications. Cells.

[35] Sidhom K., Obi P.O., Saleem A. (2020). A Review of Exosomal Isolation Methods: Is Size Exclusion Chromatography the Best Option?. Int. J. Mol. Sci..

[36] Huang J.-H., Fu C.-H., Xu Y., Yin X.-M., Cao Y., Lin F.-Y. (2020). Extracellular Vesicles Derived from Epidural Fat-Mesenchymal Stem Cells Attenuate NLRP3 Inflammasome Activation and Improve Functional Recovery After Spinal Cord Injury. Neurochem. Res..

[37] Zeng J., Gu C., Sun Y., Chen X. (2023). Engineering of M2 Macrophages-Derived Exosomes via Click Chemistry for Spinal Cord Injury Repair. Adv. Health Mater..

[38] Chen K., Yu W., Zheng G., Xu Z., Yang C., Wang Y., Yue Z., Yuan W., Hu B., Chen H. (2024). Biomaterial-based regenerative therapeutic strategies for spinal cord injury. NPG Asia Mater..

[39] Liu S., Xie Y.-Y., Wang B. (2019). Role and prospects of regenerative biomaterials in the repair of spinal cord injury. Neural Regen. Res..

[40] Tabesh H., Amoabediny G., Nik N.S., Heydari M., Yosefifard M., Siadat S.R., Mottaghy K. (2008). The role of biodegradable engineered scaffolds seeded with Schwann cells for spinal cord regeneration. Neurochem. Int..

[41] Poongodi R., Chen Y.-L., Yang T.-H., Huang Y.-H., Yang K.D., Lin H.-C., Cheng J.-K. (2021). Bio-Scaffolds as Cell or Exosome Carriers for Nerve Injury Repair. Int. J. Mol. Sci..

[42] Zhang L., Fan C., Hao W., Zhuang Y., Liu X., Zhao Y., Chen B., Xiao Z., Chen Y., Dai J. (2021). NSCs Migration Promoted and Drug Delivered Exosomes-Collagen Scaffold via a Bio-Specific Peptide for One-Step Spinal Cord Injury Repair. Adv. Health Mater..

[43] Hsu J.-M., Shiue S.-J., Yang K.D., Shiue H.-S., Hung Y.-W., Pannuru P., Poongodi R., Lin H.-Y., Cheng J.-K. (2020). Locally Applied Stem Cell Exosome-Scaffold Attenuates Nerve Injury-Induced Pain in Rats. J. Pain Res..

[44] Fan B., Chopp M., Zhang Z.G., Liu X.S. (2020). Emerging Roles of microRNAs as Biomarkers and Therapeutic Targets for Diabetic Neuropathy. Front. Neurol..

[45] Gebert L.F.R., MacRae I.J. (2019). Regulation of microRNA function in animals. Nat. Rev. Mol. Cell Biol..

[46] Treiber T., Treiber N., Meister G. (2019). Regulation of microRNA biogenesis and its crosstalk with other cellular pathways. Nat. Rev. Mol. Cell Biol..

[47] Xia S., Xu C., Liu F., Chen G. (2023). Development of microRNA-based therapeutics for central nervous system diseases. Eur. J. Pharmacol..

[48] Wang N., He L., Yang Y., Li S., Chen Y., Tian Z., Ji Y., Wang Y., Pang M., Wang Y. (2019). Integrated analysis of competing endogenous RNA (ceRNA) networks in subacute stage of spinal cord injury. Gene.

[49] Ding S.-Q., Chen J., Wang S.-N., Duan F.-X., Chen Y.-Q., Shi Y.-J., Hu J.-G., Lü H.-Z. (2019). Identification of serum exosomal microRNAs in acute spinal cord injured rats. Exp. Biol. Med..

[50] Paim L.R., Schreiber R., de Rossi G., Matos-Souza J.R., Costa E.S.A.A., Calegari D.R., Cheng S., Marques F.Z., Sposito A.C., Gorla J.I. (2019). Circulating microRNAs, Vascular Risk, and Physical Activity in Spinal Cord-Injured Subjects. J. Neurotrauma.

[51] Wang Y., Yi H., Song Y. (2022). miRNA Therapy in Laboratory Models of Acute Spinal Cord Injury in Rodents: A Meta-analysis. Cell. Mol. Neurobiol..

[52] Feng J., Zhang Y., Zhu Z., Gu C., Waqas A., Chen L. (2021). Emerging Exosomes and Exosomal MiRNAs in Spinal Cord Injury. Front. Cell Dev. Biol..

[53] Kang J., Guo Y. (2022). Human Umbilical Cord Mesenchymal Stem Cells Derived Exosomes Promote Neurological Function Recovery in a Rat Spinal Cord Injury Model. Neurochem. Res..

[54] Wang Y., Lai X., Wu D., Liu B., Wang N., Rong L. (2021). Umbilical mesenchymal stem cell-derived exosomes facilitate spinal cord functional recovery through the miR-199a-3p/145-5p-mediated NGF/TrkA signaling pathway in rats. Stem Cell Res. Ther..

[55] Sun G., Li G., Li D., Huang W., Zhang R., Zhang H., Duan Y., Wang B. (2018). hucMSC derived exosomes promote functional recovery in spinal cord injury mice via attenuating inflammation. Mater. Sci. Eng. C.

[56] Gao X., Gao L.F., Zhang Y.N., Kong X.Q., Jia S., Meng C.Y. (2023). Huc-MSCs-derived exosomes attenuate neuropathic pain by inhibiting activation of the TLR2/MyD88/NF-κB signaling pathway in the spinal microglia by targeting Rsad2. Int. Immunopharmacol..

[57] Xiao X., Li W., Rong D., Xu Z., Zhang Z., Ye H., Xie L., Wu Y., Zhang Y., Wang X. (2021). Human umbilical cord mesenchymal stem cells-derived extracellular vesicles facilitate the repair of spinal cord injury via the miR-29b-3p/PTEN/Akt/mTOR axis. Cell Death Discov..

[58] Zhang Z., Zou X., Zhang R., Xie Y., Feng Z., Li F., Han J., Sun H., Ouyang Q., Hua S. (2021). Human umbilical cord mesenchymal stem cell-derived exosomal miR-146a-5p reduces microglial-mediated neuroinflammation via suppression of the IRAK1/TRAF6 signaling pathway after ischemic stroke. Aging.

[59] Che J., Wang H., Dong J., Wu Y., Zhang H., Fu L., Zhang J. (2024). Human umbilical cord mesenchymal stem cell-derived exosomes attenuate neuroinflammation and oxidative stress through the NRF2/NF-κB/NLRP3 pathway. CNS Neurosci. Ther..

[60] Zhou W., Silva M., Feng C., Zhao S., Liu L., Li S., Zhong J., Zheng W. (2021). Exosomes derived from human placental mesenchymal stem cells enhanced the recovery of spinal cord injury by activating endogenous neurogenesis. Stem Cell Res. Ther..

[61] Cheshmi H., Mohammadi H., Akbari M., Nasiry D., Rezapour-Nasrabad R., Bagheri M., Abouhamzeh B., Poorhassan M., Mirhoseini M., Mokhtari H. (2023). Human Placental Mesenchymal Stem Cell-derived Exosomes in Combination with Hyperbaric Oxygen Synergistically Promote Recovery after Spinal Cord Injury in Rats. Neurotox. Res..

[62] Lu Y., Zhang J., Zeng F., Wang P., Guo X., Wang H., Qin Z., Tao T. (2022). Human PMSCs-derived small extracellular vesicles alleviate neuropathic pain through miR-26a-5p/Wnt5a in SNI mice model. J. Neuroinflammation.

[63] Harrell C.R., Volarevic V., Djonov V., Volarevic A. (2022). Therapeutic Potential of Exosomes Derived from Adipose Tissue-Sourced Mesenchymal Stem Cells in the Treatment of Neural and Retinal Diseases. Int. J. Mol. Sci..

[64] Xie Y., Chen Y., Zhu Y., Chen X., Lin T., Zhou D. (2021). Adipose Mesenchymal Stem Cell-Derived Exosomes Enhance PC12 Cell Function through the Activation of the PI3K/AKT Pathway. Stem Cells Int..

[65] Li M., Lei H., Xu Y., Yang B., Yu C., Yuan Y., Fang D., Xin Z., Guan R. (2018). Exosomes derived from mesenchymal stem cells exert therapeutic effect in a rat model of cavernous nerves injury. Andrology.

[66] Liang Y., Wu J.-H., Zhu J.-H., Yang H. (2022). Exosomes Secreted by Hypoxia–Pre-conditioned Adipose-Derived Mesenchymal Stem Cells Reduce Neuronal Apoptosis in Rats with Spinal Cord Injury. J. Neurotrauma.

[67] Luo Y., He Y.-Z., Wang Y.-F., Xu Y.-X., Yang L. (2023). Adipose-derived mesenchymal stem cell exosomes ameliorate spinal cord injury in rats by activating the Nrf2/HO-1 pathway and regulating microglial polarization. Folia Neuropathol..

[68] Wu S., Chen Z., Wu Y., Shi Q., Yang E., Zhang B., Qian Y., Lian X., Xu J. (2024). ADSC-Exos enhance functional recovery after spinal cord injury by inhibiting ferroptosis and promoting the survival and function of endothelial cells through the NRF2/SLC7A11/GPX4 pathway. Biomed. Pharmacother..

[69] Sheng X., Zhao J., Li M., Xu Y., Zhou Y., Xu J., He R., Lu H., Wu T., Duan C. (2021). Bone Marrow Mesenchymal Stem Cell-Derived Exosomes Accelerate Functional Recovery After Spinal Cord Injury by Promoting the Phagocytosis of Macrophages to Clean Myelin Debris. Front. Cell Dev. Biol..

[70] Lankford K.L., Arroyo E.J., Nazimek K., Bryniarski K., Askenase P.W., Kocsis J.D. (2018). Intravenously delivered mesenchymal stem cell-derived exosomes target M2-type macrophages in the injured spinal cord. PLoS ONE.

[71] Ni H., Yang S., Siaw-Debrah F., Hu J., Wu K., He Z., Yang J., Pan S., Lin X., Ye H. (2019). Exosomes Derived from Bone Mesenchymal Stem Cells Ameliorate Early Inflammatory Responses Following Traumatic Brain Injury. Front. Neurosci..

[72] Li C., Qin T., Liu Y., Wen H., Zhao J., Luo Z., Peng W., Lu H., Duan C., Cao Y. (2022). Microglia-Derived Exosomal microRNA-151-3p Enhances Functional Healing After Spinal Cord Injury by Attenuating Neuronal Apoptosis via Regulating the p53/p21/CDK1 Signaling Pathway. Front. Cell Dev. Biol..

[73] Wang L., Pei S., Han L., Guo B., Li Y., Duan R., Yao Y., Xue B., Chen X., Jia Y. (2018). Mesenchymal Stem Cell-Derived Exosomes Reduce A1 Astrocytes via Downregulation of Phosphorylated NFκB P65 Subunit in Spinal Cord Injury. Cell. Physiol. Biochem..

[74] Zhang X., Jiang W., Lu Y., Mao T., Gu Y., Ju D., Dong C. (2023). Exosomes combined with biomaterials in the treatment of spinal cord injury. Front. Bioeng. Biotechnol..

[75] Liu W., Wang Y., Gong F., Rong Y., Luo Y., Tang P., Zhou Z., Zhou Z., Xu T., Jiang T. (2019). Exosomes Derived from Bone Mesenchymal Stem Cells Repair Traumatic Spinal Cord Injury by Suppressing the Activation of A1 Neurotoxic Reactive Astrocytes. J. Neurotrauma.

[76] Fan L., Liu C., Chen X., Zheng L., Zou Y., Wen H., Guan P., Lu F., Luo Y., Tan G. (2022). Exosomes-Loaded Electroconductive Hydrogel Synergistically Promotes Tissue Repair after Spinal Cord Injury via Immunoregulation and Enhancement of Myelinated Axon Growth. Adv. Sci..

[77] Nie H., Jiang Z. (2021). Bone mesenchymal stem cell-derived extracellular vesicles deliver microRNA-23b to alleviate spinal cord injury by targeting toll-like receptor TLR4 and inhibiting NF-κB pathway activation. Bioengineered.

[78] Jiang Z., Zhang J. (2021). Mesenchymal stem cell-derived exosomes containing miR-145-5p reduce inflammation in spinal cord injury by regulating the TLR4/NF-κB signaling pathway. Cell Cycle.

[79] Jia Y., Yang J., Lu T., Pu X., Chen Q., Ji L., Luo C. (2021). Repair of spinal cord injury in rats via exosomes from bone mesenchymal stem cells requires sonic hedgehog. Regen. Ther..

[80] Xin W., Qiang S., Jianing D., Jiaming L., Fangqi L., Bin C., Yuanyuan C., Guowang Z., Jianguang X., Xiaofeng L. (2021). Human Bone Marrow Mesenchymal Stem Cell–Derived Exosomes Attenuate Blood–Spinal Cord Barrier Disruption via the TIMP2/MMP Pathway After Acute Spinal Cord Injury. Mol. Neurobiol..

[81] Li S., Liao X., He Y., Chen R., Zheng W.V., Tang M., Guo X., Chen J., Hu S., Sun J. (2022). Exosomes derived from NGF-overexpressing bone marrow mesenchymal stem cell sheet promote spinal cord injury repair in a mouse model. Neurochem. Int..

[82] Mai Z., Chen H., Ye Y., Hu Z., Sun W., Cui L., Zhao X. (2021). Translational and Clinical Applications of Dental Stem Cell-Derived Exosomes. Front. Genet..

[83] Swanson W.B., Zhang Z., Xiu K., Gong T., Eberle M., Wang Z., Ma P.X. (2020). Scaffolds with controlled release of pro-mineralization exosomes to promote craniofacial bone healing without cell transplantation. Acta Biomater..

[84] Liu C., Hu F., Jiao G., Guo Y., Zhou P., Zhang Y., Zhang Z., Yi J., You Y., Li Z. (2022). Dental pulp stem cell-derived exosomes suppress M1 macrophage polarization through the ROS-MAPK-NFκB P65 signaling pathway after spinal cord injury. J. Nanobiotechnology.

[85] Li S., Luo L., He Y., Li R., Xiang Y., Xing Z., Li Y., Albashari A.A., Liao X., Zhang K. (2021). Dental pulp stem cell-derived exosomes alleviate cerebral ischaemia-reperfusion injury through suppressing inflammatory response. Cell Prolif..

[86] Shao M., Jin M., Xu S., Zheng C., Zhu W., Ma X., Lv F. (2020). Exosomes from Long Noncoding RNA-Gm37494-ADSCs Repair Spinal Cord Injury via Shifting Microglial M1/M2 Polarization. Inflammation.

[87] Li C., Jiao G., Wu W., Wang H., Ren S., Zhang L., Zhou H., Liu H., Chen Y. (2019). Exosomes from Bone Marrow Mesenchymal Stem Cells Inhibit Neuronal Apoptosis and Promote Motor Function Recovery via the Wnt/β-catenin Signaling Pathway. Cell Transplant..

[88] Wang S., Bao L., Fu W., Deng L., Ran J. (2021). Protective effect of exosomes derived from bone marrow mesenchymal stem cells on rats with diabetic nephropathy and its possible mechanism. Am. J. Transl. Res..

[89] Fukuoka T., Kato A., Hirano M., Ohka F., Aoki K., Awaya T., Adilijiang A., Sachi M., Tanahashi K., Yamaguchi J. (2021). Neurod4 converts endogenous neural stem cells to neurons with synaptic formation after spinal cord injury. iScience.

[90] Deng M., Xie P., Chen Z., Zhou Y., Liu J., Ming J., Yang J. (2021). Mash-1 modified neural stem cells transplantation promotes neural stem cells differentiation into neurons to further improve locomotor functional recovery in spinal cord injury rats. Gene.

[91] Hu X.-Y., Li W.-Y., Zhu Q.-B., Jin L.-Y., Yang Y., Xu X.-Y. (2021). Exosomes derived from human induced pluripotent stem cell-derived neural progenitor cells protect neuronal function under ischemic conditions. Neural Regen. Res..

[92] Chen J., Zhang C., Li S., Li Z., Lai X., Xia Q. (2021). Exosomes Derived from Nerve Stem Cells Loaded with FTY720 Promote the Recovery after Spinal Cord Injury in Rats by PTEN/AKT Signal Pathway. J. Immunol. Res..

[93] Zhang L., Han P. (2022). Neural stem cell-derived exosomes suppress neuronal cell apoptosis by activating autophagy via miR-374-5p/STK-4 axis in spinal cord injury. J. Musculoskelet. Neuronal Interact..

[94] Zhong D., Cao Y., Li C.-J., Li M., Rong Z.-J., Jiang L., Guo Z., Lu H.-B., Hu J.-Z. (2020). Neural stem cell-derived exosomes facilitate spinal cord functional recovery after injury by promoting angiogenesis. Exp. Biol. Med..

[95] Xu Y., Zhu Z.-H., Xu X., Sun H.-T., Zheng H.-M., Zhang J.-L., Wang H.-H., Fang J.-W., Liu Y.-Z., Huang L.-L. (2023). Neuron-Derived Exosomes Promote the Recovery of Spinal Cord Injury by Modulating Nerve Cells in the Cellular Microenvironment of the Lesion Area. Mol. Neurobiol..

[96] Li C., Qin T., Zhao J., He R., Wen H., Duan C., Lu H., Cao Y., Hu J. (2021). Bone Marrow Mesenchymal Stem Cell-Derived Exosome-Educated Macrophages Promote Functional Healing After Spinal Cord Injury. Front. Cell. Neurosci..

[97] Peng W., Wan L., Luo Z., Xie Y., Liu Y., Huang T., Lu H., Hu J. (2021). Microglia-Derived Exosomes Improve Spinal Cord Functional Recovery after Injury via Inhibiting Oxidative Stress and Promoting the Survival and Function of Endothelia Cells. Oxidative Med. Cell. Longev..

[98] Luo Z., Peng W., Xu Y., Xie Y., Liu Y., Lu H., Cao Y., Hu J. (2021). Exosomal OTULIN from M2 macrophages promotes the recovery of spinal cord injuries via stimulating Wnt/β-catenin pathway-mediated vascular regeneration. Acta Biomater..

[99] Zhang B., Lin F., Dong J., Liu J., Ding Z., Xu J. (2021). Peripheral Macrophage-derived Exosomes promote repair after Spinal Cord Injury by inducing Local Anti-inflammatory type Microglial Polarization via Increasing Autophagy. Int. J. Biol. Sci..

[100] Peng P., Yu H., Xing C., Tao B., Li C., Huang J., Ning G., Zhang B., Feng S. (2021). Exosomes-mediated phenotypic switch of macrophages in the immune microenvironment after spinal cord injury. Biomed. Pharmacother..

[101] Pan D., Li Y., Yang F., Lv Z., Zhu S., Shao Y., Huang Y., Ning G., Feng S. (2021). Increasing toll-like receptor 2 on astrocytes induced by Schwann cell-derived exosomes promotes recovery by inhibiting CSPGs deposition after spinal cord injury. J. Neuroinflammation.

[102] Pan D., Zhu S., Zhang W., Wei Z., Yang F., Guo Z., Ning G., Feng S. (2021). Autophagy induced by Schwann cell-derived exosomes promotes recovery after spinal cord injury in rats. Biotechnol. Lett..

[103] Ren J., Zhu B., Gu G., Zhang W., Li J., Wang H., Wang M., Song X., Wei Z., Feng S. (2023). Schwann cell-derived exosomes containing MFG-E8 modify macrophage/microglial polarization for attenuating inflammation via the SOCS3/STAT3 pathway after spinal cord injury. Cell Death Dis..

[104] Yuan X., Wu Q., Wang P., Jing Y., Yao H., Tang Y., Li Z., Zhang H., Xiu R. (2019). Exosomes Derived from Pericytes Improve Microcirculation and Protect Blood–Spinal Cord Barrier After Spinal Cord Injury in Mice. Front. Neurosci..

[105] Ma K., Xu H., Zhang J., Zhao F., Liang H., Sun H., Li P., Zhang S., Wang R., Chen X. (2019). Insulin-like growth factor-1 enhances neuroprotective effects of neural stem cell exosomes after spinal cord injury via an miR-219a-2-3p/YY1 mechanism. Aging.

[106] Sun H., Cao X., Gong A., Huang Y., Xu Y., Zhang J., Sun J., Lv B., Li Z., Guan S. (2021). Extracellular vesicles derived from astrocytes facilitated neurite elongation by activating the Hippo pathway. Exp. Cell Res..

[107] Wong F.C., Ye L., Demir I.E., Kahlert C. (2021). Schwann cell-derived exosomes: Janus-faced mediators of regeneration and disease. Glia.

[108] Ghosh M., Pearse D.D. (2023). Schwann Cell-Derived Exosomal Vesicles: A Promising Therapy for the Injured Spinal Cord. Int. J. Mol. Sci..

[109] Zhu B., Gu G., Ren J., Song X., Li J., Wang C., Zhang W., Huo Y., Wang H., Jin L. (2023). Schwann Cell-Derived Exosomes and Methylprednisolone Composite Patch for Spinal Cord Injury Repair. ACS Nano.

[110] Jessen K.R., Mirsky R., Lloyd A.C. (2015). Schwann Cells: Development and Role in Nerve Repair. Cold Spring Harb. Perspect. Biol..

[111] Ching R.C., Wiberg M., Kingham P.J. (2018). Schwann cell-like differentiated adipose stem cells promote neurite outgrowth via secreted exosomes and RNA transfer. Stem Cell Res. Ther..

[112] Cong M., Shen M., Wu X., Li Y., Wang L., He Q., Shi H., Ding F. (2021). Improvement of sensory neuron growth and survival via negatively regulating PTEN by miR-21-5p-contained small extracellular vesicles from skin precursor-derived Schwann cells. Stem Cell Res. Ther..

[113] Hyung S., Kim J., Yu C., Jung H., Hong J. (2019). Neuroprotective effect of glial cell-derived exosomes on neurons. Immunotherapy.

[114] Huang Z., Guo L., Huang L., Shi Y., Liang J., Zhao L. (2021). Baicalin-loaded macrophage-derived exosomes ameliorate ischemic brain injury via the antioxidative pathway. Mater. Sci. Eng. C.

[115] Gao Z.-S., Zhang C.-J., Xia N., Tian H., Li D.-Y., Lin J.-Q., Mei X.-F., Wu C. (2021). Berberine-loaded M2 macrophage-derived exosomes for spinal cord injury therapy. Acta Biomater..

[116] Huang J.-H., He H., Chen Y.-N., Liu Z., Romani M.D., Xu Z.-Y., Xu Y., Lin F.-Y. (2022). Exosomes derived from M2 Macrophages Improve Angiogenesis and Functional Recovery after Spinal Cord Injury through HIF-1α/VEGF Axis. Brain Sci..

[117] Poulen G., Aloy E., Bringuier C.M., Mestre-Francés N., Artus E.V., Cardoso M., Perez J.-C., Goze-Bac C., Boukhaddaoui H., Lonjon N. (2021). Inhibiting microglia proliferation after spinal cord injury improves recovery in mice and nonhuman primates. Theranostics.

[118] Ge X., Guo M., Hu T., Li W., Huang S., Yin Z., Li Y., Chen F., Zhu L., Kang C. (2020). Increased Microglial Exosomal miR-124-3p Alleviates Neurodegeneration and Improves Cognitive Outcome after rmTBI. Mol. Ther..

[119] Zhang J., Hu D., Li L., Qu D., Shi W., Xie L., Jiang Q., Li H., Yu T., Qi C. (2024). M2 Microglia-derived Exosomes Promote Spinal Cord Injury Recovery in Mice by Alleviating A1 Astrocyte Activation. Mol. Neurobiol..

[120] Endo F., Kasai A., Soto J.S., Yu X., Qu Z., Hashimoto H., Gradinaru V., Kawaguchi R., Khakh B.S. (2022). Molecular basis of astrocyte diversity and morphology across the CNS in health and disease. Science.

[121] Escartin C., Galea E., Lakatos A., O’Callaghan J.P., Petzold G.C., Serrano-Pozo A., Steinhäuser C., Volterra A., Carmignoto G., Agarwal A. (2021). Reactive astrocyte nomenclature, definitions, and future directions. Nat. Neurosci..

[122] Liddelow S.A., Guttenplan K.A., Clarke L.E., Bennett F.C., Bohlen C.J., Schirmer L., Bennett M.L., Münch A.E., Chung W.-S., Peterson T.C. (2017). Neurotoxic reactive astrocytes are induced by activated microglia. Nature.

[123] Hira K., Ueno Y., Tanaka R., Miyamoto N., Yamashiro K., Inaba T., Urabe T., Okano H., Hattori N. (2018). Astrocyte-Derived Exosomes Treated With a Semaphorin 3A Inhibitor Enhance Stroke Recovery via Prostaglandin D_2_ Synthase. Stroke.

[124] Lu Y., Chen C., Wang H., Du R., Ji J., Xu T., Yang C., Chen X. (2022). Astrocyte-derived sEVs alleviate fibrosis and promote functional recovery after spinal cord injury in rats. Int. Immunopharmacol..

[125] Tang H.-B., Jiang X.-J., Wang C., Liu S.-C. (2018). S1P/S1PR3 signaling mediated proliferation of pericytes via Ras/pERK pathway and CAY10444 had beneficial effects on spinal cord injury. Biochem. Biophys. Res. Commun..

[126] Zeng X., Bian W., Liu Z., Li J., Ren S., Zhang J., Zhang H., Tegeleqi B., He G., Guan M. (2023). Muscle-derived stem cell exosomes with overexpressed miR-214 promote the regeneration and repair of rat sciatic nerve after crush injury to activate the JAK2/STAT3 pathway by targeting PTEN. Front. Mol. Neurosci..

[127] Azoulay-Alfaguter I., Mor A. (2018). Proteomic analysis of human T cell-derived exosomes reveals differential RAS/MAPK signaling. Eur. J. Immunol..

[128] Rao F., Zhang D., Fang T., Lu C., Wang B., Ding X., Wei S., Zhang Y., Pi W., Xu H. (2019). Exosomes from Human Gingiva-Derived Mesenchymal Stem Cells Combined with Biodegradable Chitin Conduits Promote Rat Sciatic Nerve Regeneration. Stem Cells Int..

[129] Tassew N.G., Charish J., Shabanzadeh A.P., Luga V., Harada H., Farhani N., D’onofrio P., Choi B., Ellabban A., Nickerson P.E. (2017). Exosomes Mediate Mobilization of Autocrine Wnt10b to Promote Axonal Regeneration in the Injured CNS. Cell Rep..

[130] He W., Zhang X., Li X., Ju D., Mao T., Lu Y., Gu Y., Qi L., Wang Q., Wu Q. (2022). A decellularized spinal cord extracellular matrix-gel/GelMA hydrogel three-dimensional composite scaffold promotes recovery from spinal cord injury via synergism with human menstrual blood-derived stem cells. J. Mater. Chem. B.

[131] Cao Y., Xu Y., Chen C., Xie H., Lu H., Hu J. (2021). Local delivery of USC-derived exosomes harboring ANGPTL3 enhances spinal cord functional recovery after injury by promoting angiogenesis. Stem Cell Res. Ther..

[132] Kong G., Xiong W., Li C., Xiao C., Wang S., Li W., Chen X., Wang J., Chen S., Zhang Y. (2023). Treg cells-derived exosomes promote blood-spinal cord barrier repair and motor function recovery after spinal cord injury by delivering miR-2861. J. Nanobiotechnology.

[133] Gao P., Yi J., Chen W., Gu J., Miao S., Wang X., Huang Y., Jiang T., Li Q., Zhou W. (2023). Pericyte-derived exosomal miR-210 improves mitochondrial function and inhibits lipid peroxidation in vascular endothelial cells after traumatic spinal cord injury by activating JAK1/STAT3 signaling pathway. J. Nanobiotechnology.

[134] Mao Q., Nguyen P.D., Shanti R.M., Shi S., Shakoori P., Zhang Q., Le A.D. (2019). Gingiva-Derived Mesenchymal Stem Cell-Extracellular Vesicles Activate Schwann Cell Repair Phenotype and Promote Nerve Regeneration. Tissue Eng. Part A.

[135] Yuan F., Peng W., Yang Y., Xu J., Liu Y., Xie Y., Huang T., Shi C., Ding Y., Li C. (2023). Endothelial progenitor cell-derived exosomes promote anti-inflammatory macrophages via SOCS3/JAK2/STAT3 axis and improve the outcome of spinal cord injury. J. Neuroinflammation.

[136] Gauthier J., Vincent A.T., Charette S.J., Derome N. (2019). A brief history of bioinformatics. Brief. Bioinform..

[137] Wei Z.-J., Feng S.-Q., Li J.-Z., Fan B.-Y., Sun T., Wang X.-X., Li J.-J., Zhang J.-P., Gu G.-J., Shen W.-Y. (2022). Bioinformatics analysis of ferroptosis in spinal cord injury. Neural Regen. Res..

[138] Hu R., Shi M., Xu H., Wu X., He K., Chen Y., Wu L., Ma R. (2022). Integrated bioinformatics analysis identifies the effects of Sema3A/NRP1 signaling in oligodendrocytes after spinal cord injury in rats. PeerJ.

[139] He X., Fan L., Wu Z., He J., Cheng B. (2017). Gene expression profiles reveal key pathways and genes associated with neuropathic pain in patients with spinal cord injury. Mol. Med. Rep..

[140] Zhang G., Yang P. (2017). Bioinformatics Genes and Pathway Analysis for Chronic Neuropathic Pain after Spinal Cord Injury. BioMed Res. Int..

[141] Dong H., Zhang C., Shi D., Xiao X., Chen X., Zeng Y., Li X., Xie R. (2022). Ferroptosis related genes participate in the pathogenesis of spinal cord injury via HIF-1 signaling pathway. Brain Res. Bull..

[142] Ge M.-H., Tian H., Mao L., Li D.-Y., Lin J.-Q., Hu H.-S., Huang S.-C., Zhang C.-J., Mei X.-F. (2021). Zinc attenuates ferroptosis and promotes functional recovery in contusion spinal cord injury by activating Nrf2/GPX4 defense pathway. CNS Neurosci. Ther..

[143] Zhou H., Yin C., Zhang Z., Tang H., Shen W., Zha X., Gao M., Sun J., Xu X., Chen Q. (2020). Proanthocyanidin promotes functional recovery of spinal cord injury via inhibiting ferroptosis. J. Chem. Neuroanat..

[144] Dixon S.J., Lemberg K.M., Lamprecht M.R., Skouta R., Zaitsev E.M., Gleason C.E., Patel D.N., Bauer A.J., Cantley A.M., Yang W.S. (2012). Ferroptosis: An Iron-Dependent Form of Nonapoptotic Cell Death. Cell.

[145] Bai X.-Y., Liu X.-L., Deng Z.-Z., Wei D.-M., Zhang D., Xi H.-L., Wang Q.-Y., He M.-Z., Yang Y.-L. (2023). Ferroptosis is a new therapeutic target for spinal cord injury. Front. Neurosci..

[146] Yao X., Zhang Y., Hao J., Duan H.-Q., Zhao C.-X., Sun C., Li B., Fan B.-Y., Li W.-X., Fu X.-H. (2019). Deferoxamine promotes recovery of traumatic spinal cord injury by inhibiting ferroptosis. Neural Regen. Res..

[147] Chavda V.P., Pandya A., Kumar L., Raval N., Vora L.K., Pulakkat S., Patravale V., Salwa, Duo Y., Tang B.Z. (2023). Exosome nanovesicles: A potential carrier for therapeutic delivery. Nano Today.

[148] Singh N., Guha L., Kumar H. (2024). From hope to healing: Exploring the therapeutic potential of exosomes in spinal cord injury. Extracell. Vesicle.

[149] Cheng L.-F., You C.-Q., Peng C., Ren J.-J., Guo K., Liu T.-L. (2024). Mesenchymal stem cell-derived exosomes as a new drug carrier for the treatment of spinal cord injury: A review. Chin. J. Traumatol..

[150] Ren Z.-W., Zhou J.-G., Xiong Z.-K., Zhu F.-Z., Guo X.-D. (2019). Effect of exosomes derived from MiR-133b-modified ADSCs on the recovery of neurological function after SCI. Eur. Rev. Med. Pharmacol. Sci..

[151] Zhang Y., Chopp M., Liu X.S., Katakowski M., Wang X., Tian X., Wu D., Zhang Z.G. (2016). Exosomes Derived from Mesenchymal Stromal Cells Promote Axonal Growth of Cortical Neurons. Mol. Neurobiol..

[152] Huang S., Ge X., Yu J., Han Z., Yin Z., Li Y., Chen F., Wang H., Zhang J., Lei P. (2018). Increased miR-124-3p in microglial exosomes following traumatic brain injury inhibits neuronal inflammation and contributes to neurite outgrowth via their transfer into neurons. FASEB J..

[153] Li H., He Y., Chen X., Yang A., Lyu F., Dong Y. (2024). Exosomal miR-423-5p Derived from Cerebrospinal Fluid Pulsation Stress-Stimulated Osteoblasts Improves Angiogenesis of Endothelial Cells via DUSP8/ERK1/2 Signaling Pathway. Stem Cells Int..

[154] Ge X., Tang P., Rong Y., Jiang D., Lu X., Ji C., Wang J., Huang C., Duan A., Liu Y. (2021). Exosomal miR-155 from M1-polarized macrophages promotes EndoMT and impairs mitochondrial function via activating NF-κB signaling pathway in vascular endothelial cells after traumatic spinal cord injury. Redox Biol..

[155] Zhang A., Bai Z., Yi W., Hu Z., Hao J. (2021). Overexpression of miR-338-5p in exosomes derived from mesenchymal stromal cells provides neuroprotective effects by the Cnr1/Rap1/Akt pathway after spinal cord injury in rats. Neurosci. Lett..

[156] Li C., Li X., Zhao B., Wang C. (2020). Exosomes derived from miR-544-modified mesenchymal stem cells promote recovery after spinal cord injury. Arch. Physiol. Biochem..

[157] Chang Q., Hao Y., Wang Y., Zhou Y., Zhuo H., Zhao G. (2021). Bone marrow mesenchymal stem cell-derived exosomal microRNA-125a promotes M2 macrophage polarization in spinal cord injury by downregulating IRF5. Brain Res. Bull..

[158] Zhou S., Ding F., Gu X. (2016). Non-coding RNAs as Emerging Regulators of Neural Injury Responses and Regeneration. Neurosci. Bull..

[159] Yang W., Sun P. (2020). Promoting functions of microRNA-29a/199B in neurological recovery in rats with spinal cord injury through inhibition of the RGMA/STAT3 axis. J. Orthop. Surg. Res..

[160] Jiang D., Gong F., Ge X., Lv C., Huang C., Feng S., Zhou Z., Rong Y., Wang J., Ji C. (2020). Neuron-derived exosomes-transmitted miR-124-3p protect traumatically injured spinal cord by suppressing the activation of neurotoxic microglia and astrocytes. J. Nanobiotechnology.

[161] Zhu J., Yang J., Xu J. (2021). miR-223 Inhibits the Polarization and Recruitment of Macrophages via NLRP3/IL-1*β* Pathway to Meliorate Neuropathic Pain. Pain Res. Manag..

[162] Bai G., Jiang L., Meng P., Li J., Han C., Wang Y., Wang Q. (2020). LncRNA Neat1 Promotes Regeneration after Spinal Cord Injury by Targeting miR-29b. J. Mol. Neurosci..

[163] Huang W., Lin M., Yang C., Wang F., Zhang M., Gao J., Yu X. (2021). Rat Bone Mesenchymal Stem Cell-Derived Exosomes Loaded with miR-494 Promoting Neurofilament Regeneration and Behavioral Function Recovery after Spinal Cord Injury. Oxidative Med. Cell. Longev..

[164] Malvandi A.M., Rastegar-Moghaddam S.H., Ebrahimzadeh-Bideskan S., Lombardi G., Ebrahimzadeh-Bideskan A., Mohammadipour A. (2022). Targeting miR-21 in spinal cord injuries: A game-changer?. Mol. Med..

[165] Ma X., Ma T., Chang L., Chen X., Xia G., Li C., Liu H. (2022). Correlation between miRNA-124, miRNA-544a, and TNF-α levels in acute spinal cord injury. Spinal Cord.

[166] Cui Y., Yin Y., Xiao Z., Zhao Y., Chen B., Yang B., Xu B., Song H., Zou Y., Ma X. (2019). LncRNA Neat1 mediates miR-124-induced activation of Wnt/β-catenin signaling in spinal cord neural progenitor cells. Stem Cell Res. Ther..

[167] Wang T., Li B., Yuan X., Cui L., Wang Z., Zhang Y., Yu M., Xiu Y., Zhang Z., Li W. (2019). MiR-20a Plays a Key Regulatory Role in the Repair of Spinal Cord Dorsal Column Lesion via PDZ-RhoGEF/RhoA/GAP43 Axis in Rat. Cell. Mol. Neurobiol..

[168] Danilov C.A., Gu Y., Punj V., Wu Z., Steward O., Schönthal A.H., Tahara S.M., Hofman F.M., Chen T.C. (2020). Intravenous delivery of microRNA-133b along with Argonaute-2 enhances spinal cord recovery following cervical contusion in mice. Spine J..

[169] Shao C., Chen Y., Yang T., Zhao H., Li D. (2022). Mesenchymal Stem Cell Derived Exosomes Suppress Neuronal Cell Ferroptosis Via lncGm36569/miR-5627-5p/FSP1 Axis in Acute Spinal Cord Injury. Stem Cell Rev. Rep..

[170] Akhlaghpasand M., Tavanaei R., Hosseinpoor M., Yazdani K.O., Soleimani A., Zoshk M.Y., Soleimani M., Chamanara M., Ghorbani M., Deylami M. (2024). Safety and potential effects of intrathecal injection of allogeneic human umbilical cord mesenchymal stem cell-derived exosomes in complete subacute spinal cord injury: A first-in-human, single-arm, open-label, phase I clinical trial. Stem Cell Res. Ther..

[171] Ye H., Wang F., Xu G., Shu F., Fan K., Wang D. (2023). Advancements in engineered exosomes for wound repair: Current research and future perspectives. Front. Bioeng. Biotechnol..

[172] Lotfy A., AboQuella N.M., Wang H. (2023). Mesenchymal stromal/stem cell (MSC)-derived exosomes in clinical trials. Stem Cell Res. Ther..

